# Identification of PLXNC1 as a novel biomarker for consensus molecular subtype 4 in colorectal cancer

**DOI:** 10.1016/j.gendis.2025.101974

**Published:** 2025-12-11

**Authors:** Weiqi Wang, Mingxuan Zhou, Tiegang Li, Wenqiang Gan, Silin Lv, Zheng Yan, Yufang Hou, Zifan Zeng, Liu Yang, Fang Zhang, Wenyi Zhao, Min Yang

**Affiliations:** a State Key Laboratory of Digestive Health, Institute of Materia Medica, Chinese Academy of Medical Sciences and Peking Union Medical College, Beijing 100050, China; b State Key Laboratory of Bioactive Substance and Function of Natural Medicines, Institute of Materia Medica, Chinese Academy of Medical Sciences and Peking Union Medical College, Beijing 100050, China

**Keywords:** Bioinformatics, Biomarker, Colorectal cancer (CRC), Consensus molecular subtype 4 (CMS4), Plexin C1 (PLXNC1), Prognosis

## Abstract

Colorectal cancer (CRC) is a highly heterogeneous malignancy that is divided into four consensus molecular subtypes (CMSs), with CMS4 considered to be the worst type of CRC. The CMS classification has not been translated into clinical practice through the transcriptomic data; thus, the identification of a novel biomarker that can identify CRC subpopulations is imperative. Using bioinformatic analysis and immunohistochemical verification, we found that PLXNC1 mRNA and protein expression were significantly up-regulated in CRC tissues. High PLXNC1 was found to be significantly associated with aggressiveness and poor prognosis and thus was confirmed as an independent prognostic factor for CRC. CMS4 CRC could be distinguished from other subtypes based on PLXNC1 expression. The tumor microenvironment was investigated by deconvolution algorithms through bulk and single-cell RNA-sequencing data. CRC with high PLXNC1 expression exhibited a distinct mesenchymal phenotype, accompanied by high infiltration of stromal components, angiogenesis, complement activation, and an immunosuppressive microenvironment. The functions of PLXNC1 were assessed by proliferation, migration, and invasion assays in CRC cells *in vitro*. A subcutaneous tumor model and liver metastasis model of CRC were generated to explore the effects of PLXNC1 *in vivo.* Combined with RNA-sequencing analysis, PLXNC1 promoted CRC growth and metastasis by regulating epithelial–mesenchymal transformation and immune escape. Knockdown of PLXNC1 reduced the CMS4-related phenotypes in CRC. *In vitro* co-culture and *in vivo* experiments revealed that PLXNC1 could impair the cytotoxicity of CD8^+^ T cells, thus facilitating immune evasion by CRC cells. We demonstrate that PLXNC1 may predict poor prognosis of CRC, exhibit pro-oncogenic effects, and accelerate tumor immune escape in CRC progression. Our study uncovers a novel biomarker for CMS4 CRC and suggests PLXNC1 as an indicator for prognosis and a potential drug target of high-risk CRC.

## Introduction

Colorectal carcinoma (CRC) is the third most common cancer, with over 1,800,000 new cases a year worldwide.^1^ Although recent advances, including molecularly targeted agents and immunotherapy, have improved outcomes, CRC remains one of the most lethal cancers, and new treatments are urgently needed. Patients with CRC often experience inconsistent outcomes and drug responses due to tumor heterogeneity.^2^ Thus, biomarkers to determine CRC subtypes are urgently needed. The identification of a correlation between the KRAS mutation and EGFR mAbs resulted in the first pre-planned biomarker therapy.^3^ However, the paradigm of one drug for one gene model resulted in disappointing efficacy, indicating the considerable complexity of tumorigenesis and drug-resistance mechanisms. Recent advances in the molecular understanding of CRC, especially the clinical application of transcriptome sequencing, enabled investigators to link gene expression patterns to homogeneous cellular phenotypes, leading to “multi-molecular, multi-drug” strategies.^4^ A large international effort led to a unified transcriptomic classification system for most CRCs that includes four consensus molecular subtypes (CMSs).^5^

Importantly, not only do the four CMS subtypes possess distinct biological attributes, but they are also associated with different clinical outcomes. Retrospective studies and clinical trials using the CMS classification led to patient stratification and targeted clinical strategies. CMS4 (mesenchymal subtype) exhibits strikingly poor outcomes and limited responses to standard adjuvant chemotherapy treatment in patients with early-stage CRC.^5^^,^^6^ In the CMS4, epithelial–mesenchymal transition (EMT), transforming growth factor-beta (TGF-β) signaling, angiogenesis, and the complement-mediated inflammatory system are excessively activated.^5^ CMS4 CRC is characterized by mesenchymal tumors with an immunosuppressive tumor microenvironment (TME) due to the integrated alterations of both cancer cells and tumor stroma.^7^ Specifically, the mesenchymal subgroup CMS4 has received more attention owing to its enhanced capacity for distant metastasis and its association with poor prognosis. Although the current treatments are ineffective, the biological interpretation of CMS4 raises hope for novel subtype-based interventions in the future. Therefore, we aimed to introduce a robust predictive biomarker for the CMS4 into the “multi-molecular, multi-drug” framework to facilitate the translation of CMS classification to clinical therapy.

Plexin C1 (PLXNC1) is a member of the plexin family composed of a transmembrane protein and an endogenous receptor of the neuronal guidance protein semaphorin 7A, which was first described as a factor mediating neuronal cell adhesion in the optic tectum.^8^ Accumulating evidence has confirmed that PLXNC1 is widely up-regulated in a variety of human cancers (*e.g.*, gastric cancer,^9^^,^^10^ hepatocellular carcinoma,^11^ non-small-cell lung cancer,^12^ papillary thyroid cancer,^13^ and acute myeloid leukemia^14^) and serves as a cancer promoter. Recent studies have also revealed that the expression abundance of PLXNC1 is markedly associated with tumor progression and even prognosis. For instance, high PLXNC1 expression is significantly correlated with aggressiveness and poor prognosis in patients with gastric cancer.^9^^,^^10^ Similarly, PLXNC1 has also been reported to be an IDO1-interacting gene and a strong predictor of survival in acute myeloid leukemia.^14^ Moreover, PLXNC1 knockdown inhibited the proliferation, colony formation, and invasiveness of papillary thyroid cancer cells, and increased cell apoptosis.^13^ PLXNC1 has a key role in inflammatory conditions, immune responses through leukocyte transmigration, and inflammatory cytokine production.^15^^,^^16^ In particular, PLXNC1 appears to be an immune-related gene, which has essential roles in promoting gastric cancer progression; this is supported by the evidence that PLXNC1 is significantly associated with M2 macrophages^9^ and enhances the IL-6/STAT3 signaling pathway.^10^ PLXNC1 has been shown to have a prominent role in the pathogenesis of immune responses and tumor development; however, little is known about the role of PLXNC1 in CRC, especially regarding its clinical significance, CMS classification, and molecular mechanisms.

In this study, we investigated the prognostic value of PLXNC1 in CRC and the critical role of PLXNC1 in inducing the mesenchymal phenotype, immunosuppressive environment, and immune escape in CRC using multifaceted bioinformatical methods and experimental validation. Our results demonstrate that PLXNC1 is a promising biomarker for identifying CMS4 CRC. Furthermore, based on our experimental results using *in vitro* and *in vivo* models combined with RNA sequencing, PLXNC1 was found to play multi-dimensional oncogenic functions in CRC, suggesting that it is a potential molecular target for treating patients with high-risk CRC.

## Methods

### Patient cohorts and specimens

We determined the mRNA expression levels of PLXNC1 in CRC using the Oncomine database^17^ with a threshold *p*-value of 0.05. After rejecting adenoma samples and DNA expression analyses, 5 over-expression and 12 under-expression analyses were included in the meta-analysis. The protein expression data of PLXNC1 in colon cancer were provided by the Clinical Proteomic Tumor Analysis Consortium (CPTAC)^18^ and are accessible in UALCAN.^19^

The clinical information, DNA methylation data, somatic mutation data, and mRNA expression data from The Cancer Genome Atlas (TCGA)-colon adenocarcinomas (COAD) and TCGA-rectal adenocarcinoma (READ) cohorts were downloaded from the GDC portal.^20^ The mRNA expression was converted from fragments per kilobase of transcript per million mapped reads (FPKM) to transcripts per million (TPM). Normal samples, samples from the same patient, and samples without overall survival (OS) information or OS days < 1 were excluded; thus, 590 samples were included in the analysis. Five Affymetrix microarray datasets (GSE39582,^21^ GSE37892,^22^ GSE17536,^23^ GSE35896,^24^ GSE41258^25^^,^^26^) were used as validation sets. The RMA-normalized gene expression and clinical information were collected from the Gene Expression Omnibus (GEO)^27^ database. The genomic annotation of PLXNC1 transcripts and methylation probes used in TCGA data was extracted from the UCSC genome browser. The DNA methylation of each probe was plotted separately. The correlation between probes and PLXNC1 expression is displayed. A total of 30 colon cancer tissues and matched normal tissues were collected from each patient and used to construct a tissue microarray (TMA). Informed consent was obtained from all of the participating patients. This work was approved by the Research Ethics Committee of The Affiliated Hospital of Qingdao University and was carried out in accordance with the 1964 Declaration of Helsinki and its later revisions.

### Survival analysis

The prognostic impact of PLXNC1 on OS was estimated using a Kaplan–Meier analysis. Patients were divided into two groups based on the optimal cut-off for PLXNC1 expression. The hazard ratios for the PLXNC1 groups and other clinical characteristics were determined using univariable Cox regression analyses. The predictors with a *p*-value < 0.05 were included in the multivariable Cox regression. The N stage and clinical stage exhibited a co-linear relationship. Therefore, only the parameter with the higher hazard ratio was included. The nomogram was plotted to predict 1-, 2-, and 3-year survival probabilities for patients with CRC. Calibration curves and receiver operator characteristic (ROC) curves were used to determine the accuracy of the Cox models. The survival analysis was performed using survminer, survival, rms, and pROC packages in R.

### Pathways analysis

An enrichment analysis of the 50 genes in the TCGA-Colon and Rectal Adenocarcinoma (COREAD) cohort with the highest co-expression with PLXNC1 was performed. The GO and KEGG analyses, including over-representation analysis and gene set enrichment analysis (GSEA),^28^ were visualized using the ClusterProfiler package with default values in R. Gene sets related to CMS4 were downloaded from the molecular signatures database. The gene set variation analysis (GSVA) score for each sample was calculated using the GSVA package and visualized with the ComplexHeatmap package in R. Stromal and immune infiltration was evaluated with the Estimation of STromal and Immune cells in MAlignant Tumor tissues using Expression data (ESTIMATE) algorithm.^29^ The immune cell composition was characterized by deconvolution from gene expression profiles. The main immune component estimation for patients from TCGA-COREAD was extracted from Cell-type Identification By Estimating Relative Subsets Of RNA Transcripts (CIBERSORT)^30^ and Tumor IMmune Estimation Resource (TIMER).^31^ The correlations between PLXNC1 and immune markers or EMT markers were analyzed using the Spearman test. The immune infiltration analysis was visualized with the ComplexHeatmap, ggstatsplot, ggcorrplot, and beanplot packages in R.

### CMS and immune subtypes

The CMS labels for each patient in TCGA-COREAD and GEO datasets were previously published.^5^ The predictive value of PLXNC1 for the CMS classification was evaluated using the ROC plots for single datasets and the sum of all samples from the four datasets in a Sankey diagram. In the Sankey plot, samples were divided into PLXNC1-Low or PLXNC1-High groups based on the median expression of PLXNC1 in each dataset. The plots were drawn using pROC and the riverplot package in R.

Immune subtypes in the TCGA pan-cancer dataset were proposed by Thorsson et al^32^ and provided in the interactive iAtlas portal. Single-nucleotide variant neoantigen, indel neoantigen, and cancer-testis antigen scores for the COREAD samples were also obtained from the iAtlas portal. Mutation and neoantigen loads were downloaded from The Cancer Immunome Atlas.^33^ The homologous recombination deficiency, loss of heterozygosity, large-scale state transitions, the number of telomeric allelic imbalances, and combined homologous recombination deficiency scores of TCGA samples were estimated by Knijnenburg et al^34^ to describe DNA damage repair alterations. The PLXNC1 expression levels in patients with C1–C6 subtypes of colorectal cancer (CRC) were compared with the correlations between PLXNC1 expression and the C6 signature. The accuracy of PLXNC1 expression, TGF-β expression, and the TGF-β signature in discriminating the C6 group in the pan-cancer cohort was evaluated with ROC curves. The mutation profiles of the PLXNC1-High and PLXNC1-Low groups were visualized with the maftools package. A logistic regression model was developed to compare the most frequently mutated genes in the different groups.

### Single-cell RNA sequence analysis

The single-cell RNA sequence data from 23 patients were downloaded from the dataset GSE132465.^35^ Data from primary CRC were processed with the Seurat package and normalized with the Harmony package in R. Using the t-distributed stochastic neighbor embedding (tSNE) technique with a resolution of 0.1, 47,285 cells were clustered into 10 clusters. The differentially expressed genes in each cluster were identified using the “FindAllMarkers” function. The clusters were annotated with Cellmarker 2.0^36^ and combined into seven clusters. Intercellular communications between different cells were predicted with the R package CellChat.^37^

### Cell culture and siRNA transfections

The LoVo, SW480, and HCT116 cell lines (human colon adenocarcinoma cell lines) were purchased from BNBIO (Beijing, China). All CRC cell lines were cultured in medium (F12K for LoVo, F12 for SW480 and HCT116, Gibco, Carlsbad, California, USA) supplemented with 10% fetal bovine serum (Gibco, Carlsbad, California, USA) and 1% penicillin-streptomycin (Gibco, Carlsbad, California, USA) in a humidified atmosphere containing 5% CO_2_ at 37 °C. For PLXNC1 knockdown, CRC cells at 50%–70% confluence were transfected with 50 nM siRNA (Cyagen Biosciences, Guangzhou, China) using a jetPRIME Polyplus kit (Polyplus Transfection Inc., Illkirch, France) for 48 h. The siRNA sequences are listed in Table S1.

### Real-time quantitative reverse transcription PCR (qRT-PCR)

Total RNA was extracted from CRC cells using a commercial RNA Tissue/Cell Rapid Extraction Kit (Shandong Sparkjade Biotechnology Co., Ltd.) and subjected to reverse transcription using a SPARKscript ll RT Plus Kit (Shandong Sparkjade Biotechnology Co., Ltd.), according to the manufacturer's instructions. A SPARKscript II SYBR One Step qRT-PCR Kit (Shandong Sparkjade Biotechnology Co., Ltd.) was used to perform the qRT-PCR reaction, and the reactions were repeated at least three times. GAPDH was used as an internal control. The primer sequences for qRT-PCR are listed in Table S2.

### Cell viability, Transwell, and proliferation assays

CRC cells (1 × 10^4^ cells/well) were seeded in 96-well plates with six replicates and transfected with siRNA or DNA. CCK-8 reagent (10 μL, DOJINDO, Kumamoto, Japan) was added to each well at 0, 24, 48, and 72 h after transfection and incubated at 37 °C for 4 h. Absorbance at 450 nm was measured using a microplate reader (PerkinElmer EnVision, Massachusetts, USA).

CRC cells (2 × 10^4^ cells/well) were seeded onto Transwell inserts (8 μm, Corning, New York, USA) coated or uncoated with Matrigel. CRC cells were transfected with siRNA or DNA for 24 h with four replicates. Medium containing 1% fetal bovine serum was added to the upper chamber, and medium with 10% fetal bovine serum was added to the bottom chamber to stimulate cell migration and invasion. After incubation for 48 h, CRC cells that passed through the membrane were fixed with 4% paraformaldehyde, stained with 0.1% crystal violet, and counted under a microscope (Nikon Eclipse, Ts2R, Nikon, Tokyo, Japan).

CRC cells (1.5 × 10^5^ cells/well) were seeded onto glass-bottom cell culture dishes (20 mm, NEST Biotechnology) and transfected with siRNA for 48 h with three replicates. Next, 50 μmol/L 5-ethynyl-2′-deoxyuridine (EdU) was incorporated into cells for 2 h. Following the manufacturer's instructions, the proliferation of CRC cells was detected using the E-Click EdU Cell Proliferation Imaging assay kit (E-CK-A377, Elabscience Biotechnology Co., Ltd., China). The cell nuclei were stained with Hoechst 33342, and images were photographed with a confocal microscope (Leica SP8X, Germany). The numbers of cells were detected by ImageJ, and the cell proliferation ratio was calculated by the ratio of the number of EdU^+^ cells to the number of Hoechst^+^ cells.

### Immunofluorescence and immunohistochemical staining

For immunofluorescence staining, LoVo cells were cultured in confocal dishes and transfected with siRNA for 48 h. After fixing with 4% paraformaldehyde, cells were permeabilized with 0.3% Triton X-100 and blocked with 5% bovine serum albumin. Fixed cells were incubated with primary antibody against E-cadherin (1:50; sc-8426; Santa Cruz Biotechnology, California, USA) or vimentin (1:100; BM0135; Boster, Wuhan, China) at 4 °C overnight, followed by incubation with secondary antibodies (1:200; Boster, Wuhan, China) at room temperature for 1 h. The nuclei of cells were stained with Hoechst for 10 min. Images were photographed with a confocal microscope (Leica SP8X, Germany).

For immunohistochemical staining, TMA or paraffin-embedded mouse tumor sections were deparaffinized, rehydrated, and incubated with 3% H_2_O_2_. Antigens were retrieved with citrate buffer at 100 °C. After blocking with 5% bovine serum albumin, the sections were incubated with primary antibodies (Table S3) at 4 °C overnight, followed by incubation with secondary antibodies (BST19013894, Boster, Wuhan, China; BST20023895, Boster, Wuhan, China) at room temperature for 1 h. The sections were stained with 3,3′-diaminobenzidine and counterstained with hematoxylin. Two pathologists, who were blinded to the study group, independently scored the sections based on staining intensity and percentage.

### Western blotting analysis

Total protein from CRC cells treated with siRNA-NC or siRNA-PLXNC1 for 48 h was collected using lysis buffer (Cwbio, Beijing, China) supplemented with protease inhibitor cocktail (Roche, Basel, Switzerland). The protein concentration was quantified through a bicinchoninic acid protein assay (Pierce Biotechnology, Rockford, Illinois, USA), after which the protein was separated by 10% sodium dodecyl sulphate-polyacrylamide gel electrophoresis and transferred onto nitrocellulose membranes (Bio-Rad, Hercules, California, USA). The membranes were incubated with specific primary antibodies against SNAIL (13099-1-AP, Proteintech, 1:500), vimentin (10366-1-AP, Proteintech, 1:10000), E-cadherin (20874-1-AP, Proteintech, 1:10000), and GAPDH (#5174, Cell Signaling, 1:1000) at 4 °C overnight, followed by incubation with the appropriate secondary antibodies (S8002, Sudgen, 1:10000). Images were obtained using an ImageQuantTM LAS 4000 luminescent image analyzer (GE, Boston, Massachusetts, USA)

### Animal studies

The animal studies were approved by the Institutional Animal Care and Use Committee of the Institute of Materia Medica, Chinese Academy of Medical Sciences. Female 8-week-old C57BL/6 mice were purchased from Vital River Laboratory Animal Technology (Beijing, China). PLXNC1-shRNA and NC-shRNA were designed and synthesized by Cyagen Biosciences (Guangzhou, China) to silence PLXNC1. SL4, a highly metastatic murine colon adenocarcinoma cell line^38^ derived from C57BL/6 mice, was used for the *in vivo* experiments as described in our previous studies.^39^^,^^40^ SL4 cells were cultured in DMEM medium (Gibco, Carlsbad, California, USA) supplemented with 10% fetal bovine serum and 1% penicillin-streptomycin. SL4 cells were transduced with the lentiviral vectors at a multiplicity of infection of 10 to achieve an ideal knockdown efficiency. The target cells were harvested after 48 h, and 10^7^ cells/mL were resuspended in saline. The shRNA target sequences are listed in Table S4. For the implanted subcutaneous tumor model, SL4 cells (10^6^ cells in 100 μL) were injected into the left hind leg of C57BL/6 mice (*n* = 9 per group). Tumor growth *in vivo* was evaluated using a PharmaScan 70/16 US (7.0 T, Bruker, Switzerland) MRI scanner on day 14. The tumor volume was calculated as the sum of the tumor area in each slice multiplied by slice thickness using Radiant software. Mice were sacrificed on day 14 after injection, and tumors were dissected and weighed.

In the liver metastasis model of colon cancer, mice were anesthetized, and a laparotomy was performed. SL4 cells (10^6^ cells in 100 μL) were injected into the spleen (*n* = 6/7 per group). SL4 cells metastasized to the liver in all of the mice after intrasplenic injection, as previously described.^38^ Liver metastasis was estimated on day 10 by the bioluminescence signal intensity using an IVIS spectrum CT system (Caliper Life Sciences, Alameda, California, USA). Mice were intraperitoneally injected with 3 mg of D-Luciferin (Caliper Life Sciences, Alameda, California, US), which can be oxidized by luciferase-labeled SL4 cells. The signal intensity was quantified using LivingImage software (Xenogen Corp., Alameda, California, USA). Magnetic resonance images of mice were obtained on the same day. Mice were sacrificed on day 10 after surgery, and the livers were collected after cardiac perfusion. Liver weights were recorded to estimate metastasis severity.

### RNA sequencing

Total mRNA was enriched using Oligo(dT) magnetic beads and fragmented. The mRNA was reverse transcribed using random primers. After purification, the cDNA was repaired, tailed with dA, and ligated with sequence adaptors. The cDNA size distribution pattern was screened using AMPure XP Beads. Filtered cDNA was amplified by PCR to construct the library. RNA sequencing was performed on an Illumina NovaSeq 6000 (Illumina, San Diego, California, USA) by Beijing Expandbio Science & Technology. Raw data were filtered with Trimmomatic and aligned to the reference genome with STAR. Gene expression was normalized as FPKM by DESeq. The raw sequence data have been deposited in the Gene Expression Omnibus with accession number GSE261942 (https://www.ncbi.nlm.nih.gov/geo/query/acc.cgiacc=GSE261942). The criteria for differentially expressed genes were set as *p*-value < 0.05 and |log_2_ fold change| > 1. GO enrichment was determined using Goseq and visualized with Goplot. The GSEA plot was generated with clusterprofiler and enrichplot. The abundance of immune cells in tumor tissue was estimated by MMCPCOUNTER,^41^ quanTIseq,^42^ and EPIC.^43^

### CD8^+^ T cell isolation and co-culture with colon cancer cells

CD8^+^ T cells from the spleens of C57BL/6 mice were isolated using the EasySep mouse CD8^+^ T cell isolation kit (Cat 19853, Stem Cell Technologies). Briefly, 2 × 10^6^ cells/mL CD8^+^ T cells were mixed with FITC-CD8a (BD Bioscience; 553031; 1:500) for 30 min at room temperature in the dark. After washing with phosphate-buffered saline, filtering, and centrifuging, the supernatant was discarded, and stained cells were analyzed by Image-Stream MarkII imaging flow cytometry. The results were analyzed by IDEAS statistical image analysis software (Amnis, EMD-Millipore, Seattle, Washington, USA).

CD8^+^ T cells were resuspended at 1 × 10^6^ cells/mL in 1 mL RPMI culture medium containing 5 μg/mL CD3e (553058, BD Bioscience), 5 μg/mL CD28 (553295, BD Bioscience), 5 μg/mL of mouse recombinant IL-2 (Eg0456, Proteintech), and 50 μM 2-mercaptoethanol at 37 °C for 2 days. Then, the activated CD8^+^ T and SL4 cells were co-cultured for 48 h. The supernatant was aspirated and centrifuged at 300 g for 10 min. The lower layer of CD8^+^ T cells was used for RNA extraction, reverse transcription, and qRT-PCR, while the supernatant was utilized for an ELISA of IFN-γ (EMC102a, QuantiCyto). The SL4 cells in the culture dish were fixed with 4% paraformaldehyde, stained with crystal violet, and then photographed under a microscope.

### Flow cytometry

After coculture with sh-NC and sh-PLXNC1 SL4 cells, CD8^+^ T cells were harvested and incubated with Fc receptor blocking antibody CD16/32 (65057-1-Ig, Proteintech) at 4 °C for 30 min. The CD8^+^ T cells were stained with APC anti-mouse CD25 (101909, BioLegend), PE anti-mouse CTLA4 (106305, BioLegend), and PE anti-mouse Tim-3 (134003, BioLegend) at 4 °C for 30 min. After washing with phosphate-buffered saline, filtering, and centrifuging, the supernatant was discarded, and stained cells were analyzed by Image-Stream MarkII imaging flow cytometry. The results were analyzed by IDEAS statistical image analysis software (Amnis, EMD-Millipore, Seattle, Washington, USA).

### Statistical analysis

PLXNC1 expression levels in TMA-paired samples were compared using the Wilcoxon signed-rank test. The log-rank test was used for survival analysis. Correlation coefficients in heatmaps were determined by Spearman's test. The default options were used to adjust the *p*-value in the function enrichment analysis tools. Pairwise comparisons between more than two groups were performed using Kruskal–Wallis and Dunn tests. The discriminative power of PLXNC1 was assessed with ROC curves and AUC (area under the ROC curve) values. Other data were analyzed using Student's *t*-tests (normally distributed variables) or Wilcoxon rank-sum tests (non-normally distributed variables). All statistical analyses were performed using R (Version 3.6.3). The significance threshold was set at 0.05.

## Results

### Elevated PLXNC1 predicts worse outcomes in CRC

**Figure 1:**
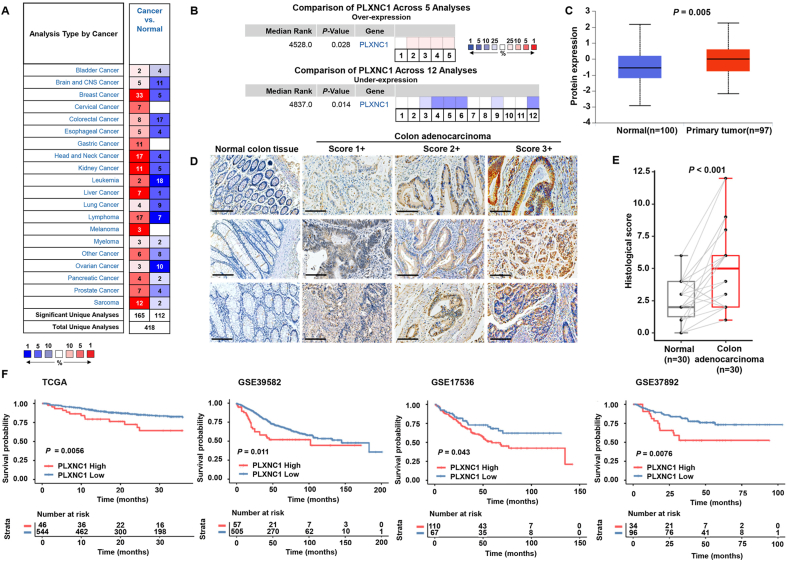
PLXNC1 is elevated and predicts worse outcomes in colorectal cancer. **(A)** PLXNC1 expression in different cancers and normal tissues in the Oncomine database. The red and blue denote up-regulation and down-regulation of PLXNC1 in the tumor tissues, respectively. **(B)** The meta-analyses of PLXNC1 over-expression cohorts or under-expression cohorts in colorectal cancer. **(C)** The protein levels of PLXNC1 in colon cancer samples and matched normal samples from the Clinical Proteomic Tumor Analysis Consortium. **(D)** Representative immunohistochemical staining for PLXNC1 based on the tissue microarray (TMA) of colon cancer samples and matched normal samples. Scale bars = 200 μm. **(E)** Quantitative analysis of PLXNC1 protein expression scores from the immunohistochemical staining of the tissue microarray. **(F)** Kaplan–Meier curves for overall survival (OS) between the high- and low-PLXNC1 expressing CRC patients from the TCGA and different GEO data sets.

Given the crucial roles of plexins in immune-related diseases, especially cancer, we conducted univariate Cox analysis of patients with colon cancer from TCGA. PLXNC1 had the highest hazard ratio (Table S5), which indicates that high expression of PLXNC1 is a risk factor for poor prognosis in patients. Subsequently, differential expression analyses using the Oncomine database show significant PLXNC1 dysregulation in CRC. As shown in Figure 1A, PLXNC1 expression increased significantly in CRC tumor tissue compared with expression in normal tissue in 8 datasets; the opposite trend was detected in the other 17 datasets. The adenoma datasets and DNA expression datasets were excluded, and the rest of the over-expression or under-expression datasets were included in the meta-analysis (Fig. 1B). Based on these initial results, PLXNC1 protein levels were investigated in the CPTAC dataset. As shown in Figure 1C, PLXNC1 protein levels were higher in the primary tumor tissue compared with the protein levels in normal tissue (*p* = 0.005). These findings were verified using tissue microarrays containing paired samples from 30 patients with colon cancer (Fig. 1D). PLXNC1 protein levels were significantly higher in CRC tumor tissue compared with protein levels in normal adjacent tissue (*p* < 0.001) (Fig. 1E).

Gene expression is usually linked to promoter methylation. Therefore, the potential mechanism for the overexpression of PLXNC1 in CRC samples was investigated. Twenty-eight methylation probes are in or near the PLXNC1 transcript, and 11 of the probes are in CpG islands. Probe methylation was compared with PLXNC1 expression (Fig. S1). Only a few negative correlations were observed, and none of these correlations were in the promoter probes (Fig. S1). Based on these results, promoter methylation was ruled out as the mechanism regulating the overexpression of PLXNC1 in CRC.

To evaluate whether the overexpression of PLXNC1 can predict outcomes, we divided TCGA-COREAD patients into two groups according to the optimal cut-off value. The Kaplan–Meier curves showed that patients with high PLXNC1 expression had worse OS (Fig. 1F). These findings were validated in the three GEO datasets. The OS was worse in patients with high PLXNC1 expression in all validation sets (Fig. 1F). The prognosis value of PLXNC1 was assessed using Cox regression. The univariate Cox model for TCGA-COREAD cohort revealed that age (*p* = 0.013), M stage (*p* < 0.001), T stage (*p* < 0.001), stage (*p* < 0.001), PLXNC1 expression (*p* = 0.007), and preoperative CEA (*p* = 0.001) were significantly associated with poor outcome (Fig. S2A). Thus, these parameters were included in the multivariate analysis, which revealed that PLXNC1 was an independent risk factor for OS in TCGA-COREAD (*p* = 0.039) (Fig. S2B) and GSE39582 (*p* = 0.017) cohorts (Figs. S2C and S3D). The nomogram provided a quantitative visualization of the Cox regression; Figure S3A and S3B show the nomogram for calculating the predicted 1-, 2-, and 3-year survival of patients with CRC using the six predictors identified in the univariate analysis. The performance of the multivariate model was assessed with the concordance index and ROC curves. The calibration curves revealed good agreement between the predicted survival and actual observations (Fig. S3C and S3D). Based on the ROC curves, our Cox model was superior to the current TNM stage (Fig. S3E and S3F).

### PLXNC1 correlates with CMS4 CRC characteristics

We next examined the relationship between PLXNC1 expression and clinical characteristics of patients with CRC using the TCGA-COREAD dataset. As shown in Figure S4A, the expression level of PLXNC1 was significantly associated with MSI status and CMS subtypes. Likewise, the 50 most co-expressed genes with PLXNC1 were identified in the TCGA-COREAD dataset (Fig. S4A). Based on GO and KEGG analyses, these 50 genes were mainly enriched in pathways related to immune response (Fig. S4B). Meanwhile, GSEA analysis revealed that the gene sets involved in biological features of CMS4 CRC, including EMT, inflammatory response, angiogenesis, TGF-β signaling, and complement, were predominantly enriched in patients with CRC with high PLXNC1 expression (Fig. S4C). GSEA further confirmed these findings and presented that PLXNC1 expression was strongly positively correlated with the CMS4 CRC signature (Fig. S4D). These results suggest a link between PLXNC1 overexpression and CMS4 CRC.

### PLXNC1 is a biomarker for CMS4 CRC

Patients classified with CMS4 CRC have mesenchymal tumors with altered immune function in the TME, resulting in the worst outcomes of the four CMS groups. The CRC Subtyping Consortium (CRCSC) provided an R package (CMSclassifier) consisting of 693 discriminant genes to classify the CMS subtype of a single sample. The potential ability of PLXNC1 to distinguish the CMS4 in CRC was investigated.

**Figure 2:**
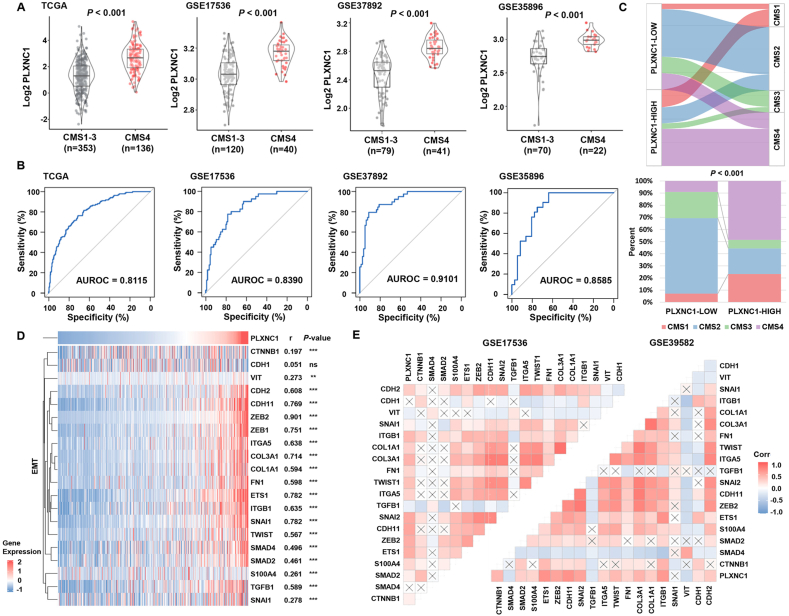
PLXNC1 is a biomarker for CMS4 CRC and is related to epithelial–mesenchymal transition. **(A)** PLXNC1 expression in CMS1–3 and CMS4 of patients with CRC from TCGA and different GEO data sets. **(B)** ROC curves to evaluate the predictive value of PLXNC1 expression in distinguishing CMS4 from CMS1–3. AUROC, area under the receiver operating characteristic. **(C)** The distribution of CMS subtypes in PLXNC1-High and PLXNC1-Low groups, as defined by the median expression. **(D)** Correlation between PLXNC1 and the epithelial–mesenchymal transition (EMT) gene signatures in the TCGA-COREAD cohort. ∗∗∗*p* < 0.001; ∗∗*p* < 0.01; ns, not significant. **(E)** PLXNC1 positively correlates with the EMT gene signatures in GEO cohorts.

An examination of the expression of PLXNC1 in the four CMS groups revealed that the expression of PLXNC1 was high in the CMS4 in the four datasets (Fig. 2A). Next, the ability of PLXNC1 to identify patients with CMS4 CRC was assessed. The ROC curves showed that PLXNC1 was an accurate and stable biomarker for CMS4 in the four independent datasets (Fig. 2B). Patients from the four datasets were categorized into PLXNC1-High and PLXNC1-Low groups based on the median value of each dataset. The proportion of CMS subtypes in the PLXNC1-High and PLXNC1-Low groups was visualized with a Sankey diagram. The PLXNC1-High group had the highest percentage of patients with CMS4, while the PLXNC1-Low group had a low percentage of patients with CMS4. Similarly, most of the patients with CMS4 CRC belonged to the PLXNC1-High group (Fig. 2C).

The immune-altered phenotype is widely observed in various cancer types. Thorsson et al^32^ identified six distinct immune subtypes in pan-cancer; the C6 subtype has the highest TGF-β signature, which is relevant to the CMS4 in CRC.^44^ In this context, we hypothesized that PLXNC1 is associated with the C6 subtype in CRC, even in the pan-cancer cohort. In patients with CRC, PLXNC1 expression was higher in the C6 subtype compared with the other subtypes (Fig. S5A). PLXNC1 strongly correlated with the TGF-β signature, the most distinctive feature of the C6 subtype (Fig. S5B and S5C). Given the limited C6 samples in CRC, the discriminate power of PLXNC1, TGF-β expression, and TGF-β signature in distinguishing C6 subtype in pan-cancer samples was compared (Fig. S5D). PLXNC1 outperformed the other two indicators, suggesting a connection between PLXNC1 and the C6 immune subtype across cancer types.

To clarify the relationship between PLXNC1 and CMS4 CRC, we next assessed the correlation of PLXNC1 expression with mesenchymal markers using multiple cohorts. The heatmap in Figure 2D shows the expression pattern of key genes implicated in EMT. The up-regulation of most EMT genes correlated with increased PLXNC1 expression in the TCGA-COREAD dataset**.** Positive correlations were also observed between PLXNC1 and EMT genes in the validation datasets (Fig. 2E).

### PLXNC1 promotes immune escape

**Figure 3:**
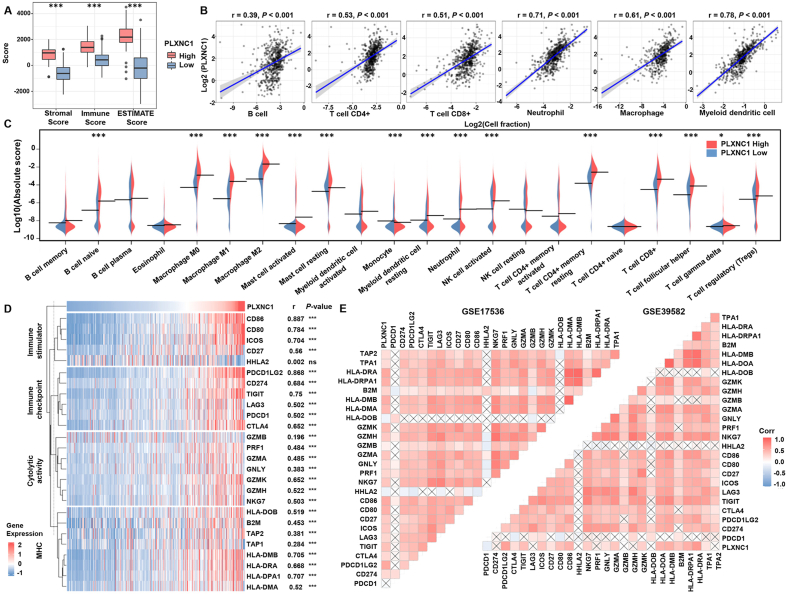
Immune landscape analysis based on the PLXNC1 expression level in patients with colorectal cancer. **(A)** Differences in immune and stromal scores between PLXNC1-High and PLXNC1-Low groups using the ESTIMATE algorithm. **(B)** Correlations between PLXNC1 expression and immune cell fractions, estimated by TIMER. **(C)** The abundances of immune cells in PLXNC1-High and PLXNC1-Low groups, estimated by the CIBERSORT algorithm. **(D)** Correlation of PLXNC1 expression with gene expression profiles of immuno-stimulator signature, immune checkpoint genes, cytolytic activity signature, and MHC family genes in TCGA-COREAD cohort. **(E)** PLXNC1 positively correlates with the immunosuppressive gene signatures in GEO cohorts. ∗∗∗*p* < 0.001 and ∗*p* < 0.05.

The TME mainly consists of extracellular matrix and molecules, blood and lymphatic vessels, stromal cells, and immune cells. We focused on stromal and immune cells, as the influence of these two components has been well documented.^45^ Based on the published gene signatures, stromal and immune scores were increased in CRC with high PLXNC1 expression (Fig. 3A). The role of immune cells in TME is complicated; thus, several deconvolution methods were used to calculate the fraction of immune cells in TCGA-COREAD patients. Figure 3B shows the correlation between PLXNC1 expression and six immune cell types. PLXNC1 strongly correlated with the neutrophil (*r* = 0.71, *p* < 0.001), macrophage (*r* = 0.61, *p* < 0.001), and myeloid dendritic cell (*r* = 0.78, *p* < 0.001) fractions. Figure 3C shows a more detailed analysis of immune cells. Although it seems contradictory, both immunosuppressive cells, including macrophage M2 and Tregs, and anti-tumor immune cells, including CD8^+^ T cells and NK cells, were elevated in the PLXNC1-High group. Although patients with high anti-tumor cells are expected to have favorable outcomes, several studies suggest that a subgroup of patients has a poor prognosis despite high CD8^+^ T cell infiltration.^46^^,^^47^ The immunosuppressive TME may severely hamper the anti-tumor cytotoxic cells via the immune and TGF-β pathways.^48^^,^^49^ As shown in Figure 3D, PLXNC1 exhibited a strong positive correlation with the multigene signatures of immune stimulators and immune checkpoints in the TCGA-COREAD dataset, with a similar co-expression pattern of cytolytic factors and MHC family molecules observed. Consistent with TCGA findings, the same trends were revealed using the two independent cohorts from GEO data sets (Fig. 3E). These results support a disorganized anti-tumor immune response in PLXNC1-High CRC.

The impact of neoantigens on the immune response was also investigated. A high neoantigen load may enhance immune recognition. Therefore, patients with a high neoantigen load may respond better to immunotherapy.^50^^,^^51^ Mutation loads, DNA damage repair events, and estimated neoantigens were based on previous studies.^32^, ^33^, ^34^ Most mutated genes were not different between the PLXNC1-High and PLXNC1-Low groups (Fig. S6A and S6B). However, the mutation burden and predicted neoantigens were slightly elevated in the PLXNC1-High group (Fig. S6C). This small increase in neoantigens is not likely to induce a dramatic change in immune infiltration. Thus, we inferred that TME alteration was the major culprit for immune escape in PLXNC1-High CRC.

### Single-cell RNA sequencing analysis shows that stroma cells with up-regulated expression of PLXNC1 exhibit increased communication with tumor cells in patients with CMS4 CRC

**Figure 4:**
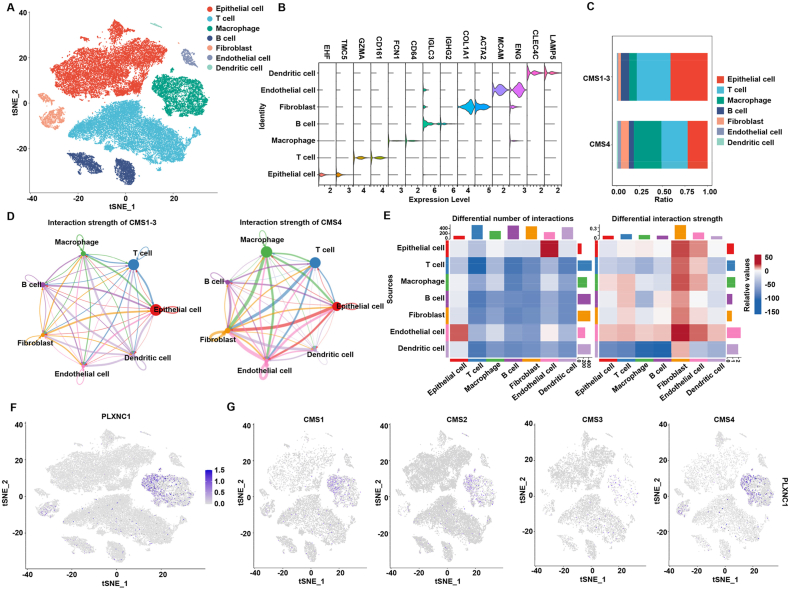
The single-cell RNA sequencing analysis reveals diverse cell types and the distribution of PLXNC1 in the four CMS subtypes. **(A)** tSNE visualization of 47285 cells from 23 tumor samples (GSE132465). **(B)** Violin plots showing specific gene markers in each cell type. **(C)** The compositions of the major cell components in different CMS subtypes. **(D)** Possible interaction strengths between the seven major cell types in CMS1–3 and CMS4 samples. **(E)** Heatmap of the difference in the communicational probability among cells. Red (positive values) and blue (negative values) indicate higher numbers of predicted interactions in CMS4 and CMS1–3, respectively. **(F)** tSNE visualization of the distribution of PLXNC1 in all samples. **(G)** tSNE visualization of the distribution of PLXNC1 in the four CMS subtypes.

To further explore the relationship between different cell types and PLXNC1, CRC single-cell RNA sequence data were extracted from GSE132465. After processing and normalization, 47285 cells from 23 tumor samples were clustered into seven cell clusters and visualized by t-distributed stochastic neighbor embedding (tSNE) (Fig. 4A). The seven cell clusters were annotated as epithelial cells (EHF, TMC5), T cells (GZMA, CD161), macrophages (FCN1, CD64), B cells (IGLC3, IGHG2), fibroblasts (COL1A1, ACTA2), endothelial cells (MCAM, ENG), and dendritic cells (CLEC4C, LAMP5) (Fig. 4B). Patients in the CMS4 group exhibited an increased proportion of macrophages and fibroblasts (Fig. 4C), in accordance with the mesenchymal phenotype,^52^ and a decreased proportion of epithelial cells, T cells, and B cells compared with the cell types in patients in the CMS1–3.

To illustrate the effect of PLXNC1 on the interaction between various cells in CRC, we performed cell-to-cell communication in the CMS1–3 and CMS4 groups using CellChat. The number and strength of interaction analyses demonstrated that tumor cells had stronger interactions with fibroblasts and endothelial cells in the CMS4 group relative to the CMS1–3 group (Fig. 4D and E). Interestingly, the tSNE results of all patients with CRC showed that PLXNC1 was predominantly expressed in macrophages and fibroblasts (Fig. 4F). PLXNC1 expression of fibroblasts was significantly increased in the CMS4 group compared with other CMS groups. These results indicate that patients with CMS4 CRC exhibit a higher level of stromal cell infiltration, including fibroblasts and endothelial cells. PLXNC1 was also found to be markedly expressed in the fibroblasts and showed a strong interaction with tumor cells in patients with CMS4 CRC.

### PLXNC1 is essential for maintaining the malignant phenotype in CRC cells

**Figure 5:**
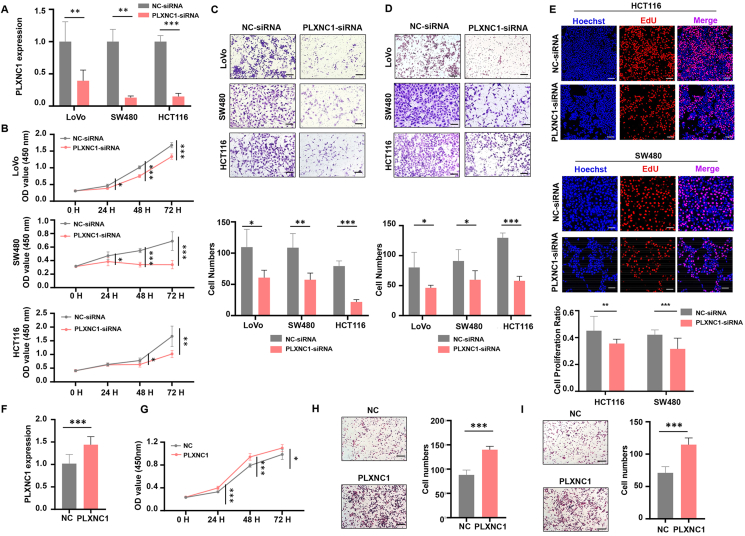
PLXNC1 promotes the proliferation, migration, and invasion of colorectal cancer (CRC) cells *in vitro.***(A)** qRT-PCR analysis was conducted to examine the knockdown efficiency of PLXNC1 in the negative control (NC)-siRNA- or PLXNC1-siRNA-transfected CRC cells. NC-siRNA was used as a negative control. GAPDH was a normalization control. **(B)** CCK-8 assay was performed to assay the viability of control and PLXNC1-silenced CRC cells. **(C)** After 48 h of transfection with NC-siRNA or PLXNC1-siRNA, the migration of CRC cells in Transwell chambers was determined. **(D)** After 48 h of transfection with NC-siRNA or PLXNC1-siRNA, CRC cells invading the Matrigel in Transwell chambers were performed. Note: Scale bars = 200 μm. **(E)** Representative images of EdU-stained (red) cells and the quantification of cell proliferation ratio normalized to Hoechst-stained (blue) cells. Scale bars = 75 μm. **(F)** qRT-PCR data of the PLXNC1 expression in LoVo cells following transfection with PLXNC1 plasmids or controls. GAPDH was a normalization control. **(G)** CCK-8 assay results show the viability of LoVo cells following transfection with PLXNC1 plasmids or controls. **(H, I)** Transwell assay was employed to count the number of migrated (H) and invaded (I) LoVo cells following transfection with PLXNC1 plasmids or controls. ∗∗∗*p* < 0.001; ∗∗*p* < 0.01, and ∗*p* < 0.05.

PLXNC1 expression was silenced in SW480, LoVo, and HCT116 cells using siRNA (Fig. 5A). PLXNC1 knockdown suppressed the proliferation of CRC cells *in vitro* (Fig. 5B). Activation of EMT pathways was linked to PLXNC1 in the TCGA cohort, and the mesenchymal phenotype promotes CRC metastasis to distant organs. Therefore, we assayed tumor invasion and distal metastasis of CRC cells using Transwell chambers. PLXNC1 knockdown markedly decreased the migration and invasion of CRC cells (Fig. 5C and D). A lower rate of EdU-stained cells further indicated that the knockdown of PLXNC1 resulted in a decrease in CRC cell proliferation rates (Fig. 5E). Furthermore, PLXNC1 was overexpressed by transfection with plasmids carrying the gene of PLXNC1 in LoVo cells (Fig. 5F). PLXNC1 overexpression enhanced the proliferation, migration, and invasion of LoVo cells (Fig. 5G–I). These *in vitro* studies suggest that PLXNC1 is a potential target for CRC therapy.

**Figure 6:**
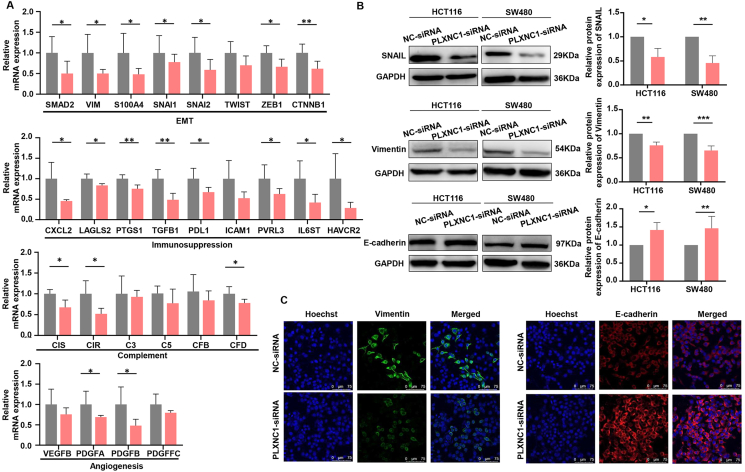
PLXNC1 knockdown inhibits gene signatures characteristic of CMS4 colorectal cancer. **(A)** qRT-PCR analysis was applied to examine the relative mRNA expression of genes related to epithelial–mesenchymal transition (EMT), complement, angiogenesis, and immunosuppression. GAPDH was a normalization control. **(B)** Protein expression and quantification results of SNAIL, Vimentin, and E-cadherin in colorectal cancer cells were determined by western blotting. **(C)** Immunofluorescence analysis of the levels of E-cadherin (the epithelial marker, red) and Vimentin (the mesenchymal marker, green) proteins in the control and PLXNC1-silenced LoVo cells. The nuclei were stained with Hoechst (blue). Scale bars = 75 μm ∗∗∗*p* < 0.001, ∗∗*p* < 0.01, and ∗*p* < 0.05.

As mentioned previously, high PLXNC1 expression is associated with mesenchymal and immunosuppressive phenotypes. To validate the molecular basis of the cell phenotype, qRT-PCR and immunofluorescence were performed in CRC cells. We found that PLXNC1 knockdown decreased the mRNA expression of mesenchymal markers (Fig. 6A), in line with the less aggressive behavior of LoVo cells. Moreover, PLXNC1 knockdown in LoVo cells reduced angiogenesis and complementary factors; immunosuppressive factors that restrict the anti-tumor activity of cytotoxic cells also declined (Fig. 6A).

Our results show that the protein levels of SNAIL decreased after PLXNC1 knockdown (Fig. 6B). Furthermore, PLXNC1 knockdown up-regulated the expression of E-cadherin (epithelial marker) and down-regulated the expression of vimentin (mesenchymal marker) compared with the corresponding controls detected by western blotting and immunofluorescence analysis (Fig. 6B and C). Immunohistochemical staining showed an increase in SNAIL and a decrease in GSK3β in the tumors of patients in the PLXNC1-shRNA group; this indicates a suppressed EMT process and reduced tumor cell invasion (Fig. S7A). These data suggest that PLXNC1 contributes to mesenchymal features in CRC cells and may induce an immunosuppressive status in the TME.

### PLXNC1 knockdown inhibits CRC growth and metastasis in murine models

**Figure 7:**
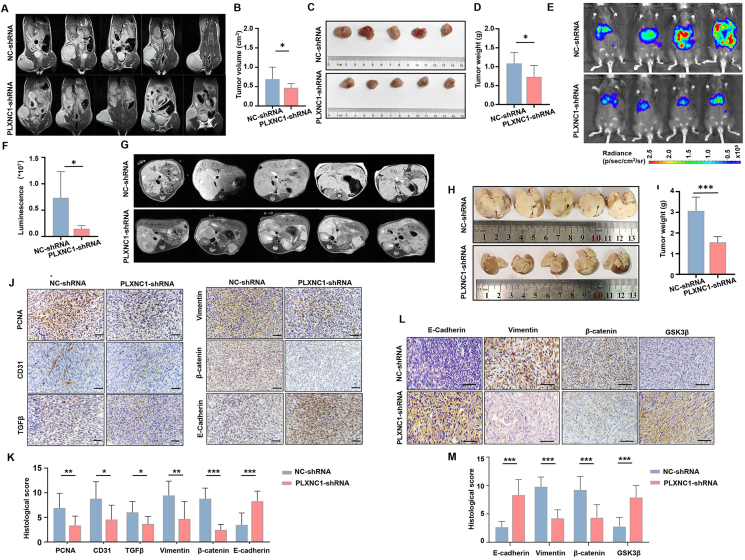
PLXNC1 promotes tumor growth and metastasis *in vivo*. **(A)** Magnetic resonance imaging (MRI) assessment of wild-type (WT) mice carrying subcutaneous tumors. **(B)** Tumor volumes calculated by MRI. The tumor volume = area of tumor in each slice × slice thickness. **(C)** Representative images of tumors from WT mice after subcutaneous inoculation of SL4 cells infected with the control vector or sh-PLXNC1 vector. **(D)** Mice were killed 14 days following subcutaneous injection, and tumors were dissected and weighed. **(E)** Bioluminescence images showing tumor metastasis by tracking luciferase-expressing SL4 cells in sh-NC and sh-PLXNC1 groups. **(F)** Histogram showing the bioluminescent signal intensity analyzed by the IVIS System. **(G)** MRI inspection of hepatic tumor metastasis of colon cancer after intrasplenic injection of SL4 cells in sh-NC and sh-PLXNC1 groups. **(H)** Gross examination of hepatic tumor metastasis of colon cancer after intrasplenic injection of SL4 cells in sh-NC and sh-PLXNC1 groups. **(I)** Mice were sacrificed on day 10 after intrasplenic injection, and livers were excised and weighed. **(J)** Immunohistochemical staining was used to detect the expression levels of PCNA, CD31, TGF-β, vimentin, β-catenin, and E-Cadherin in tumor tissues from sh-NC and sh-PLXNC1 groups. Scale bars = 50 μm. **(K)** Quantification of immunohistochemical staining in subcutaneous tumor tissues from sh-NC and sh-PLXNC1 groups. **(L)** Immunohistochemical staining showing expression of epithelial–mesenchymal transition (EMT) markers in liver metastasis tumor tissues from sh-NC and sh-PLXNC1 groups. **(M)** Quantification of immunohistochemical staining in liver metastasis tumor tissues from sh-NC and sh-PLXNC1 groups. ∗∗∗*p* < 0.001, ∗∗*p* < 0.01, and ∗*p* < 0.05; ns, not significant.

The essential role of PLXNC1 in CRC progression was confirmed *in vivo*. PLXNC1 expression was lost in SL4 cells by specific shRNAs with a lentivirus system. The effect of PLXNC1 silencing at mRNA and protein levels was measured by qRT-PCR and immunohistochemistry (Fig. S7A and S7B). SL4 cells were subcutaneously injected into the left legs of mice. After 14 days, tumor-bearing mice were examined with MRI, followed by the removal of tumors for subsequent analyses (Fig. 7A and B). The size and weight of CRC tumors were reduced by PLXNC1 knockdown in mice (Fig. 7C and D).

The liver is the most common metastatic site for CRC. A liver metastasis model was established to simulate the hematogenous spread of CRC cells through the portal veins to the liver. SL4 cells transfected with PLXNC1-shRNA or NC-shRNA were injected into the spleens of mice, and liver metastasis was estimated with image analysis. Bioluminescence imaging exhibited a significant decrease in liver metastatic foci by PLXNC1-shRNA transfection compared with the corresponding control (Fig. 7E and F). MRI images showed multiple hepatic tumor nodules in the control group, but liver metastases were significantly ameliorated by the knockdown of PLXNC1 (Fig. 7G). Gross inspection demonstrated a marked increase in liver weight in mice injected with SL4 cells after NC-shRNA transfection, whereas livers from mice injected with PLXNC1 knockdown cells presented fewer tumor foci and decreased tumor-occupied liver weight relative to that in the control group (Fig. 7H and I).

To elucidate the mechanisms underlying reduced tumor growth and metastasis upon PLXNC1 knockdown, we performed immunohistochemical analysis of key functional markers in subcutaneous tumors and liver metastases. In subcutaneous tumors (Fig. 7J and K), sh-PLXNC1 significantly down-regulated proliferation marker PCNA, indicating impaired tumor cell replication and angiogenesis indicator CD31, confirming suppressed neovascularization. Mesenchymal markers Vimentin, nuclear β-catenin, and EMT-transcription factor SNAIL significantly reduced in the PLXNC1 knockdown group (Fig. S8A and S8B). Concurrently, epithelial marker E-cadherin and GSK3β were elevated. In liver metastases, PLXNC1 deficiency similarly attenuated Vimentin expression (Fig. 7L and M) and β-catenin expression. E-cadherin and GSK3β concomitantly elevated in the sh-PLXNC1 group. Meanwhile, the relative mRNA expression of Vimentin, SNAIL and FGF7 were down-regulated in the PLXNC1 knockdown group (Fig. S9). Collectively, these data demonstrate that PLXNC1 ablation targets tumor proliferative capacity and metastatic plasticity across CRC progression stages.

### PLXNC1 deficiency reverses immunosuppression and blocks mesenchymal phenotype across primary and metastatic CRC

**Figure 8:**
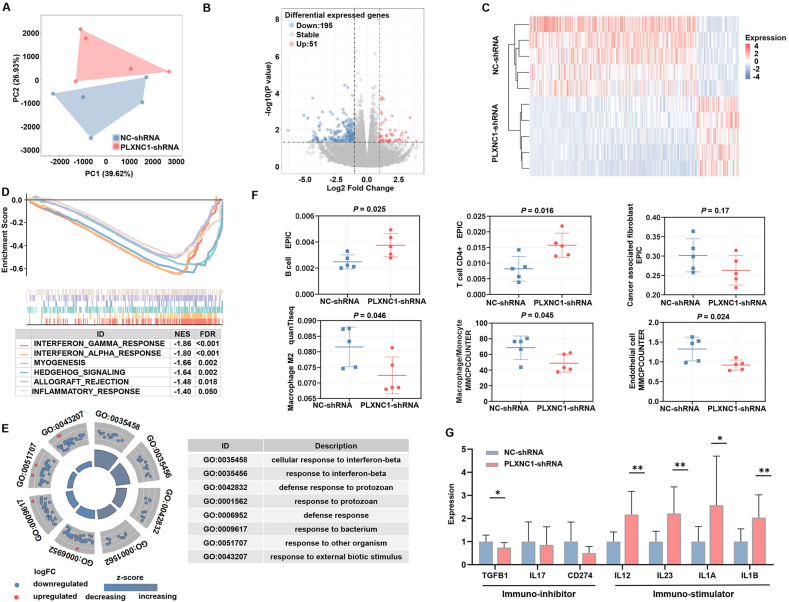
PLXNC1 deficiency reverses the immunosuppressive microenvironment and blocks the mesenchymal phenotype of tumor cells in colorectal cancer. **(A)** The principal component analysis (PCA) plots for the distribution of samples based on RNA-sequencing data from subcutaneous tumors in the sh-NC and sh-PLXNC1 groups. **(B)** Volcano plots of differentially expressed genes between the sh-NC and sh-PLXNC1 groups. **(C)** Heatmap of differentially expressed genes between the sh-NC and sh-PLXNC1 groups. **(D)** GSEA enrichment plots for tumor tissues in the sh-PLXNC1 group. **(E)** GO enrichment of differentially expressed genes between the sh-NC and sh-PLXNC1 groups. **(F)** Diverse deconvolution algorithms were performed to evaluate the immune infiltration in tumor tissues from the sh-NC and sh-PLXNC1 groups. **(G)** qRT-PCR was conducted to examine the relative mRNA levels of immuno-inhibitor or immuno-stimulator molecules in tumor tissues from the sh-NC and sh-PLXNC1 groups. ∗∗*p* < 0.01 and ∗*p* < 0.05.

RNA sequencing was used to chart the ecosystems of the subcutaneous tumors in the sh-NC and sh-PLXNC1 groups. Sample clustering was significantly different between groups and consistent within groups (Fig. 8A). After PLXNC1 knockdown, 246 differentially expressed genes were identified (Fig. 8B and C). Interferon (IFN)-γ leads to the loss of effector functions on CD8^+^ T cells by accelerating the gene expression of immune resistance pathways, including PD-1/PD-L1 and IDO1^53^, ^54^, ^55^**.** GSEA demonstrated that the gene sets involved in the IFN-γ response and Hedgehog signaling, the signaling pathway responsible for promoting tumor progression, were predominantly down-regulated in tumor tissues from the sh-PLXNC1 group (Fig. 8D). In GO enrichment analysis, differentially expressed genes were predominantly involved in immune response (Fig. 8E). To further verify whether PLXNC1 expression closely correlated with immune cell infiltration, we performed an immune profile analysis between the two groups by deconvolution algorithms. Interestingly, we found that PLXNC1 knockdown improved the TME by changing the ratio of immune cells with different functions (Fig. 8F). CD4^+^ T cells and B cells were elevated in PLXNC1 knockdown tumors, and anti-tumor components tended to increase. Cancer-associated fibroblasts, which stimulate inflammation and angiogenesis in mesenchymal CRC, decreased in PLXNC1 knockdown tumors. The decreased endothelial cells indicated that angiogenesis in mesenchymal CRC may be reduced, thereby restricting blood supply and stroma remodeling to facilitate anti-tumor activity. Accordingly, PLXNC1 knockdown disrupted the balance between immune stimulators and suppressors in the TME (Fig. 8G).

To further investigate the role of PLXNC1 in CRC liver metastasis, we analyzed RNA-Seq data from the GSE41258 dataset. A total of 47 liver metastasis tissues were stratified into PLXNC1-low and PLXNC1-high groups based on the mean expression level of PLXNC1. Differential expression analysis identified 1127 significantly dysregulated genes between the two groups (Fig. S10A). GO and KEGG analyses of liver metastasis datasets revealed robust enrichment in immune activation pathways, particularly T cell cytotoxicity, leukocyte migration, and antigen presentation (Fig. S10B and S10C). Concurrently, cancer-progression pathways including NF-κB signaling and cell–matrix adhesion were co-enriched, suggesting interplay between tumor-intrinsic oncogenic programs and immune microenvironment remodeling. GSEA revealed that PLXNC1-high liver metastases were significantly enriched in immunosuppressive programs alongside pro-tumorigenic pathways (Fig. S10D). Conversely, PLXNC1-low samples exhibited enrichment in transcriptional regulation and developmental processes, with notable suppression of oncogenic drivers (Fig. S10E). In parallel, we employed multiple algorithms in TIMER to calculate correlations between PLXNC1 expression and immune cell infiltration. In CRC liver metastases, cytotoxic lymphocytes, particularly NK cells, showed a strong negative correlation with PLXNC1 (Fig. S10F). Conversely, immunosuppressive cells such as M2 macrophages exhibited a positive correlation. Concordantly, radar plots revealed increased macrophage infiltration and diminished NK cell infiltration in PLXNC1-high tumors (Fig. S10G). Moreover, PLXNC1 expression positively correlated with immunosuppressive markers (*e.g.*, KDR, TGFBR1) and negatively correlated with immunostimulators (*e.g.*, HHLA2, TNFRSF13B) (Fig. S10H). Collectively, these data demonstrate that elevated PLXNC1 remodels the metastatic immune landscape by suppressing cytotoxic activity while promoting immunosuppressive networks, synergizing with co-enriched oncogenic pathways to drive immune evasion.

### PLXNC1 hampers the cytotoxic CD8^+^ T cells' killing function against CRC cells

**Figure 9:**
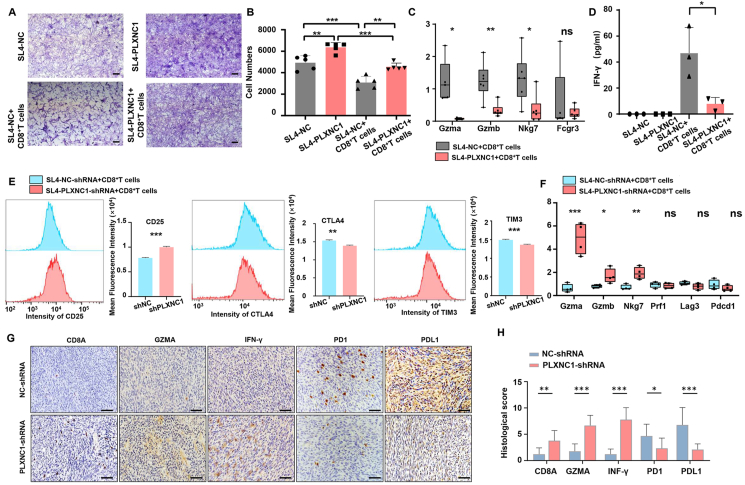
PLXNC1 hampers the colorectal cancer cell killing function of cytotoxic CD8^+^ T cells. **(A)** Representative images of crystal violet staining after coculture of SL4 cells with CD8**^+^** T cells with or without PLXNC1 overexpression. Decreased staining intensity indicates SL4 cells killed by CD8**^+^** T cells. Scale bars = 100 μm. **(B)** Quantification of remaining SL4 cells after coculture with CD8**^+^** T. **(C)** The relative mRNA expression of CD8**^+^** T cell cytotoxicity-related genes detected by qRT-PCR analysis. **(D)** The quantitative analysis of effector cytokine IFN-γ from activated CD8**^+^** T cells was identified by an ELISA kit. **(E)** Flow cytometric analysis of CD8**^+^** T cell surface markers after co-culture with control or PLXNC1-knockdown SL4 cells. **(F)** qRT-PCR analysis of mRNA expression levels of cytotoxic effector molecules and other immune-related factors in CD8**^+^** T cells following co-culture with sh-NC or sh-PLXNC1 SL4 cells. **(G)** Immunohistochemical staining showing expression of immune markers in subcutaneous tumor tissues from sh-NC and sh-PLXNC1 groups. **(H)** Quantification of immunohistochemical staining in subcutaneous tumor tissues from sh-NC and sh-PLXNC1 groups. Scale bars = 50 μm ∗∗∗*p* < 0.001, ∗∗*p* < 0.01, and ∗*p* < 0.05; ns, not significant.

To further investigate the relationship between PLXNC1 and the CMS4 subtype, which was characterized by immune evasion, we conducted co-culture experiments of CRC cells and CD8^+^ T cells (Fig. S11A). CD8^+^ T cells were isolated from the mouse spleen (Fig. S11B) and, after 48 h of activation, were co-cultured with SL4 cells with PLXNC1 overexpression for 48 h. The crystal violet staining results illustrated that PLXNC1 overexpression promoted the survival of CRC cells in the co-culture system (Fig. 9A and B). Subsequently, ELISA and qRT-PCR of CD8^+^ T cells were conducted to investigate whether PLXNC1 influences the killing effects of CD8^+^ T cells. The relative mRNA expression of the cytotoxicity-related genes, GZMA, GZMB, and NKG7, were down-regulated after co-culture with PLXNC1-overexpressed cancer cells (Fig. 9C). The levels of the effector cytokine IFN-γ in the supernatant of co-culture system was decreased in the PLXNC1 overexpressed group compared with that in the NC group (Fig. 9D). Accordingly, after cocultured with PLXNC1 knockdown SL4, CD8^+^ T cells have lower expression level of CD25 and higher expression level of TIM3 and CTLA4, which imply an immune activation phenotype (Fig. 9E). Moreover, the relative mRNA expression level of GZMA, GZMB, and NKG7 in CD8^+^ T were up-regulated with the knockdown of PLXNC1 (Fig. 9F). Additionally, immunohistochemical staining of subcutaneous (Fig. 9G and H) and liver metastasis (Fig. S12A and S12B) tumor tissues from *in vivo* studies showed that PLXNC1 knockdown significantly increased the level of CD8A and decreased the level of PDL1, which indicates the accumulation of CD8^+^ T cells. The up-regulation of GZMA and IFN-γ in the shRNA-PLXNC1 group suggests enhanced cytotoxic activity of CD8^+^ T cells. Moreover, the decrease in PD1 on the CD8^+^ T cells and PDL1 on the tumor cells in the PLXNC1-knockdown group indicates that PLXNC1 is associated with the killing function of CD8^+^ T cells. Taken together, the co-culture experiments with PLXNC1 overexpression and knockdown and the *in vivo* tumor of sh-PLXNC1 experiments demonstrate that PLXNC1 reduces the tumor-killing function of CD8^+^ T cells and promotes an immune-evasive microenvironment.

Finally, we provide a schematic diagram showing the clinical significance and biological role of PLXNC1 in CRC by using bioinformatic analysis, a real-world cohort, and experimental validation *in vitro* and *in vivo* (Fig. S13).

## Discussion

In the last decade, the heterogeneity of CRC and the association of CRC subtypes with patient survival and drug sensitivity have been extensively elucidated.^56^ New classifications were suggested for effective disease management. The CMS system includes four subtypes with distinct molecular clinical features.^5^ Of note, the CMS revealed that the composition and interaction of the TME may predict patient outcomes and responses to therapies. However, the original CMS classification is heavily reliant on genome-wide RNA analysis and 693 gene classifiers. More concise classifiers are urgently needed to advance CMS classification to a routine clinical test. In the present study, we elucidate that PLXNC1 is a surrogate marker for the CMS4, and high PLXNC1 expression is associated with the mesenchymal phenotype and an inflammatory, stromal-activated, and immunosuppressive microenvironment based on the integration of various bioinformatic approaches and experimental verification.

We demonstrated that PLXNC1 expression is elevated in CRC tissue compared with adjacent normal tissue. High PLXNC1 expression is associated with reduced survival times in all patient cohorts. Multivariable analysis revealed that PLXNC1 is an independent risk factor for OS time. Our findings are consistent with previous studies of other cancer types, including gastric cancer,^10^ non-small cell lung cancer,^12^ papillary thyroid cancer,^13^ and acute myeloid leukemia.^14^ Furthermore, PLXNC1 marks liver cancer cells with an epithelial phenotype and is significantly up-regulated in hepatocellular carcinoma.^11^ In the present study, we first reported the clinical significance and molecular function of PLXNC1 in CRC. These results suggest that PLXNC1 has oncogenic activity, which promotes CRC progression. Thus, PLXNC1 may contribute to the risk stratification of CRC patients.

In addition to the prognostic value, PLXNC1 is a potential biomarker for CMS4 CRC. CMS4 CRC is delineated by an abundant stromal infiltration, predominantly consisting of fibroblasts, which facilitates EMT and mesenchymal gene expression.^5^^,^^57^ Nevertheless, CMS4 is characterized by lower responses to adjuvant therapy and a higher incidence of metastasis and thus has the worst prognosis among the four CMS subtypes.^58^ Our enrichment analyses were the first to indicate an association between PLXNC1 and CMS4 CRC. CRC samples in the high-PLXNC1 group were markedly enriched in pathways related to CMS4 tumors, including EMT, TGF-β, and complement activation. Comparing CMS groups in the four cohorts revealed that patients classified into the CMS4 group exhibited higher PLXNC1 expression. The ROC curves show that CMS4 CRC can be distinguished from the other subtypes based on PLXNC1 expression. Thus, PLXNC1 is a potential biomarker for CMS4 CRC.

Since the CRCSC formulated the CMS subtypes, several studies aimed to simplify the classification model to facilitate clinical translation. Morris et al cut the original model containing 693 genes to 472 genes. The simplified model by Morris is more than 80% consistent with the original model. In addition, the requirements for sample storage and RNA quality are relatively low for this simplified model, making it suitable for Clinical Laboratory Improvement Amendments-certified laboratories.^59^ Li et al established a CMS classification model using only FRMD6, ZEB1, HTR2B, CDX2, and β-catenin. This model is based on immunohistochemical staining of paraffin-embedded CRC samples and exhibits 71.4% concordance with the original model.^60^ A similar model consisting of FRMD6, ZEB1, HTR2B, CDX2, and KER was proposed by Trinh et al; the concordance of this model reached 87% and exhibited good robustness in multiple validation datasets.^61^ Another research team used deep learning algorithms to establish an image recognition CMS model based on hematoxylin-eosin staining. Hematoxylin-eosin staining is routine for patients with CRC; thus, this model does not require additional tests and is suitable for remote pathological diagnosis of CRC.^62^

We propose PLXNC1 as a potential biomarker for CMS4 CRC. As a single-gene biomarker, the impact of coefficients in multiple-gene panels between different sequencing platforms is avoided. PLXNC1 can be detected by qRT-PCR or IHC, which is convenient for clinical translation. The area under the curve values of using PLXNC1 to detect CMS4 CRC reached 0.81–0.91; thus, PLXNC1 is highly accurate and specific. PLXNC1 is more accurate for distinguishing CMS1/4 from CMS2/3 than it is for distinguishing CMS4 from other CMS types. However, patients with CMS1 CRC can be readily identified based on MMR or MSI clinical tests, which are already standard of care. Thus, the overall accuracy of PLXNC1 in recognizing CMS4 CRC may be further improved simply by combining it with an already commonly used clinical assay.

We confirmed the relationship between PLXNC1 and CMS4 phenotypes. CMS4 CRC is characterized by mesenchymal tumors and an immunosuppressive TME with stromal infiltration, angiogenesis, and TGF-β activation. EMT promotes malignant behaviors in CRC tumors and leads to poor outcomes.^63^ We demonstrated that PLXNC1 is co-expressed with mesenchymal marker genes in CRC samples. The expression of mesenchymal marker genes decreased when PLXNC1 was knocked down in cell lines and mouse models, demonstrating the vital role of PLXNC1 in maintaining the mesenchymal phenotype of CRC.

The TME is a complex milieu of non-tumor cells, including stromal cells and immune cells. The components of the TME are crucial to the development of CMS4 tumors. For example, serrated adenoma (harboring BRAF^V600E^ mutations) can only progress to CMS4 tumors when TGF-β levels in the TME are high; otherwise, the adenoma will progress to a CMS1 tumor with a good prognosis.^64^ A tumor purity analysis suggests that PLXNC1-high tumors have reduced proportions of cancer cells and a corresponding higher fraction of stromal and immune cell infiltration; the fractions correspond with PLXNC1 expression. High-PLXNC1 tumors exhibit a high density of cancer-associated fibroblasts and endothelial cells in the microenvironment. Increasing stromal cells in the high-PLXNC1 group promotes tumor progression via the formation of mechanical barriers that prevent the entry of cytotoxic cells and the production of angiogenic and immunosuppressive chemokines that inhibit the activity of cytotoxic immune cells.

Immune cell infiltration was examined using various deconvolution algorithms in high-PLXNC1 tumors. Strikingly, the immune cells consisted of both immune-active and immune-suppressive cells. Generally, tumors infiltrated with CD8^+^ T cells and NK cells are associated with favorable outcomes.^65^ However, the anti-tumor activity of cytotoxic cells can be restricted by inflammatory and immunosuppressive activity. Inflammation induces immunosuppressive cells, including Tregs, myeloid-derived suppressor cells, tumor-associated macrophages, and tumor cells themselves, to produce chemokines and cytokines. These inflammatory signals reduce the anti-tumor immune response while creating a harsher suppressive infiltrate. We observed that High-PLXNC1 tumors were infiltrated by both anti-tumor and pro-tumor immune cells, yet the stroma surrounding the tumor segregated cytotoxic cells from the tumor parenchyma, and the immunosuppressive TME weakened the cytotoxic activity of the immune cells. Despite the high percentage of CD8^+^ T cells in the tumor microenvironment, cancer cells can escape from immune attack due to the immunosuppressive function of PLXNC1. This finding agrees with our earlier study showing that a subgroup of CRC exhibits poor outcomes despite high CD8^+^ T cell infiltration.^40^ In the single-cell RNA sequencing analysis, PLXNC1 was detected in the macrophages and fibroblasts of CMS4 CRC, which has a strong interaction with tumor cells in patients with CMS4 CRC. Interestingly, these two cell types were also found in higher proportions in CMS4 CRC and induced EMT.^52^ Given the high intratumor heterogeneity in CRC, our study may be useful for risk stratification and developing a novel biomarker for cancer immunotherapy.

To further investigate the biological role of PLXNC1 in CRC, we performed loss-of-function and gain-of-function studies *in vitro*, as well as in a subcutaneous tumor model and liver metastasis model of CRC combined with transcriptome sequencing. These results demonstrated for the first time that PLXNC1 contributed to the proliferation, migration, and invasion of CRC cells *in vitro* and promoted tumor development *in vivo.* Importantly, PLXNC1 knockdown alleviated EMT and mesenchymal gene expression in CRC. Furthermore, in both CRC cell lines and subcutaneous tumor models, PLXNC1 silencing induced beneficial changes in the TME, including restricted angiogenesis, the elevation of anti-tumor immune cells, and the reduction of immunosuppressive factors. The bioinformatics prediction combined with the experimental validation suggests that PLXNC1 is a promising biomarker for identifying CMS4 CRC and is a potential molecular target for CRC treatments.

The present study extends these findings to a liver metastasis model that more closely recapitulates the clinical metastatic microenvironment. Our results indicate that PLXNC1 promotes invasion, migration, and colonization of the liver parenchyma by enhancing the EMT capacity of tumor cells themselves, contributing to liver metastasis of CRC. Consistent with RNA-seq analyses from the subcutaneous tumor model, PLXNC1 knockdown suppressed mesenchymal phenotypes and down-regulated the expression of EMT-related markers. On the other hand, PLXNC1 overexpression creates an immunosuppressive microenvironment by recruiting M2 macrophages and inhibiting the infiltration of anti-tumor immune cells such as NK cells. Using the subcutaneous tumor model and the liver metastasis model of CRC, these results lead to the discovery of PLXNC1 as a powerful driver of CRC development via facilitating EMT and conferring immune evasion.

The CMS4 subtype exhibits a profoundly immunosuppressive microenvironment that attenuates CD8^+^ T cell cytotoxicity, driving poor clinical outcomes in CRC.^5^^,^^7^^,^^66^ Our findings position PLXNC1 as a pivotal orchestrator of this immune-evasion phenotype. In the co-culture of CRC cells with CD8^+^ T cells, PLXNC1 overexpression attenuated the killing effect of cytotoxic CD8^+^ T cells and reduced the levels of effector cytokines, including IFN-γ, GZMB, GZMA, and NKG7. Notably, in CD8^+^ T cell co-culture systems, PLXNC1 knockdown in CRC cells up-regulated cytotoxic molecules and reduced exhaustion markers on CD8^+^ T cells. Furthermore, *in vivo* studies indicated that knockdown of PLXNC1 could facilitate CD8^+^ T cell infiltration, suppress the expression of immune checkpoints, and elevate cytotoxic molecule production. While prior studies implicated PLXNC1 in tumor angiogenesis, its role in immune evasion remained unexplored. Here, the critical role of PLXNC1 in immune evasion of CRC has been demonstrated for the first time. This establishes PLXNC1 as both a biomarker for immune-cold tumors and a therapeutic target to overcome immunotherapy resistance.

Our work is limited by the inherent weakness of retrospective studies. We analyzed the predictive value of PLXNC1 in several datasets from different laboratories and sequencing platforms. However, multicenter prospective cohort studies are needed to validate our results. The accuracy of biomarkers is also affected by standardized laboratory processes. Thus, future studies should unify sample collection, storage, and extraction to facilitate clinical transformation.

In conclusion, our study demonstrates that PLXNC1 is up-regulated and associated with poor outcomes in patients with CRC. Furthermore, PLXNC1 is a strong predictor of CMS4 CRC; patients with high PLXNC1 expression exhibit a mesenchymal phenotype and an immunosuppressive TME. PLXNC1 plays a vital role in the immune escape-promoting effect in CRC. These findings demonstrate that PLXNC1 can serve as a novel biomarker for risk stratification and CMS classification, and it may represent a potential molecular target for treating the poor-prognosis subtype of CRC.

## CRediT authorship contribution statement

**Weiqi Wang:** Writing – original draft, Investigation, Conceptualization. **Mingxuan Zhou:** Writing – original draft, Visualization, Methodology. **Tiegang Li:** Visualization, Methodology. **Wenqiang Gan:** Methodology, Data curation. **Silin Lv:** Software, Methodology. **Zheng Yan:** Methodology, Formal analysis. **Yufang Hou:** Validation. **Zifan Zeng:** Visualization. **Liu Yang:** Validation. **Fang Zhang:** Visualization. **Wenyi Zhao:** Software. **Min Yang:** Writing – review & editing, Supervision, Funding acquisition, Conceptualization.

## Ethics declaration

Experiment protocols were approved by the Institutional Animal Care and Use Committee of the Institute of Materia Medica, Chinese Academy of Medical Sciences, and Peking Union Medical College. The studies involving human participants were reviewed and approved by the Research Ethics Committee of The Affiliated Hospital of Qingdao University (ethics approval number: QDU-HEC-2023177). Informed consents were obtained from all the participating patients. The studies were conducted in accordance with the local legislation and institutional requirements.

## Data availability

All data generated or analyzed during this study are included in this published article, and the RNA-seq data have been deposited in the GSE261942 (https://www.ncbi.nlm.nih.gov/geo/query/acc.cgi acc = GSE261942).

## Funding

This work was supported by the CAMS Innovation Fund for Medical Sciences (CIFMS) (No. 2023-I2M-2-009) and the Natural Science Foundation of China (NSFC) (No. 81773750).

## Conflict of interests

The authors declared no competing interests.
