# Supplementary material for: Identification of PLXNC1 as a novel biomarker for consensus molecular subtype 4 in colorectal cancer
Source: Genes Dis. 2025 Dec 11;13(4):101974. doi: 10.1016/j.gendis.2025.101974 (PMC13090551; doi:10.1016/j.gendis.2025.101974)
Supplement: Multimedia component 1 [file mmc1.docx]

**SUPPLEMENTARY MATERIALS**

**Supplementary Table 1：siRNA sequences**

| siRNA | siRNA sequence |
| --- | --- |
| PLXNC1-siRNA F | GAAACAACUCUUGCAUGUAAATT |
| PLXNC1-siRNA R | UUUACAUGCAAFAFUUFUUUCTT |
| Negative control-siRNA F | UUCUCCGAACGUGUCACGUTT |
| Negative control-siRNA R | ACGUGACACGUUCGGAGAATT |

**Supplementary Table 2：The primer sequences for q-RT PCR**

| **Gene** | **Primer sequence** | **Gene** | **Primer sequence** |
| --- | --- | --- | --- |
| GAPDH F | CTGACTTCAACAGCGACACC | C1S F | CTATGGGCTCTACACACGGG |
| GAPDH R | TGAGCTTGA CAAAGTGGTCGT | C1S R | GGCTGGTGGGATGTATCTGG |
| PLXNC1 F | GGTCTGGTCCCCATTGAAGG | C1R F | CGTCTGCCCGTAGCTAATCC |
| PLXNC1 R | TTGGGCAACTCTCCTACCCT | C1R R | GCAAAAACGCCCCCACTATC |
| SMAD2 F | GCGGGAGCTTAGTAATGGGG | C3 F | AGGAGGACGAATGCCAAGAC |
| SMAD2 R | TGACAGCCAATGTTCAGCCT | C3 R | GACAACCATGCTCTCGGTGA |
| VIM F | GCAGTTTTTCAGGAGCGCAA | C5 F | ACGCTGAGCTGGTAAAAGGA |
| VIM R | TCTTGTAGGAGTGTCGGTTGT | C5 R | TGTTGTGTCTCTAGGCCAGT |
| S100A4 F | TCTTGGTTTGATCCTGACTGCT | CFB F | ATCAGGCCCATTTGTCTCCC |
| S100A4 R | TCACCCTCTTTGCCCGAGTA | CFB R | TGTTGCTGGCAAGTGGTAGT |
| SNAI1 F | TAGAGTCTGAGATGCCCCGA | CFD F | TGAAGGTCAGGGTCACCCAA |
| SNAI1 R | AAATTGCCCGGGAAACAGGT | CFD R | GACCAACCAGATGCAGGAGT |
| SNAI2 F | GCGCATGCTCCATTGTCTTA | VEGFB F | AAGGAGACAGCCTTCTGTGG |
| SNAI2 R | AAAAGGCACTTGGAAGGGGTA | VEGFB R | CAGAGAGGCAGGGGTAGTGA |
| TWIST F | TTCAAAGAAACAGGGCGTGG | PDGFA F | CCGCAGTGCACACCTAGAAT |
| TWIST R | CGTCTGGGAATCACTGTCCA | PDGFA R | GGCACACCAACAACACAGAC |
| ZEB1 F | CCCACTAGGAACAGGAACCAC | PDGFB F | CTCTGTCTGGCGGGAAGAAG |
| ZEB1 R | CCCAACTTATGCCAGGCACC | PDGFB R | AGAGACACGCACAAGTCCAC |
| CTNNB1 F | TTGGAACCTTGTTTTGGACAGT | PDGFC F | TTTGGGGAAATCTGAGCCTAGC |
| CTNNB1 R | AAGCATCGTATCACAGCAGGT | PDGFC R | CACAGCACAGCACGCTTTAT |
| CXCL2 F | ACAGTGTGTGGTCAACATTTCT | ICAM1 F | GGCCCCACAGACTTACAGAA |
| CXCL2 R | CTCTGCTCTAACACAGAGGGAA | ICAM1 R | GTCAGGAAGTGTGGGCCTTT |
| LGALS1 F | CGCTAAGAGCTTCGTGCTGA | PVRL3 F | CACGTGCCTAGTAGGGTTCT |
| LGALS2 R | CGTTGAAGCGAGGGTTGAAG | PVRL3 R | CTCCTACAGCTCCTTAATGCAA |
| PTGS1 F | GGCAATCAGACACCCTCTCC | IL6ST F | GGAAGCTCAGCCAACTCGAA |
| PTGS1 R | ACCATCTTGTCAGAGCCAGC | IL6ST R | CCCAAGCAGCCTTTCCATGA |
| TGFB1 F | TGCCCATCGTCTACTACGTG | HAVCR2 F | CTACTGCTGCCGGATCCAAA |
| TGFB1 R | TTGCAGGAGCGCACAATCAT | HAVCR2 R | GTCCCCTGGTGGTAAGCATC |
| CD274 F | GGAAATTCCGGCAGTGTACC | GAPDH-M F | TGGCATTGTGGAAGGGCTCAT |
| CD274 R | TGACAGCTGGTGGCATTCAA | GAPDH-M R | CAGCTTTCCAGAGGGGCCAT |
| TGFB1-M F | ACCAACTATTGCTTCAGCTCCA | PLXNC1-M F | GAGTCATGCTGGGACAGGCT |
| TGFB1-M R | GAAGTTGGCATGGTAGCCCT | PLXNC1-M R | GCCTCTACCCTACCTTAGCTCT |
| IL12-M F | CTCATCTCCCCAAAGGTGGC | IL17-M F | TCGTGTTTGTTTTTCTGTGGACT |
| IL12-M R | ACGTCTTCGCCCCTTAACAA | IL17-M R | CCCCATTCAGAGGAGAGAACC |
| IL23-M F | CAGTACTCCAGACAGCAGCTC | CD274-M F | AAGACGAGCATAGCCGAACC |
| IL23-M R | ATATGCAGCTTTGTCACAGGTC | CD274-M R | TGTGTTGCCCAACCCGATATT |
| IL1A-M F | ACGAAGCTCTCCGTACATTCC | IL1B-M F | GGGACATTAGGCAGCACTCTC |
| IL1A-M R | CAGACAGCTTTAAGGACGGGA | IL1B-M R | CAGTGCGGGCTATGACCAAT |
| GZMA-M F | TGCTGCCCACTGTAACGTG | GZMB-M F | CCACTCTCGACCCTACATGG |
| GZMA-M R | GGTAGGTGAAGGATAGCCACAT | GZMB-M R | GGCCCCCAAAGTGACATTTATT |
| NKG7-M F | CTCTCTGGGCCTGACTTCTTC | FCGR3-M F | CAGAATGCACACTCTGGAAGC |
| NKG7-M R | TGTGTCACATGGATATAACCTGC | FCGR3-M R | GGGTCCCTTCGCACATCAG |

Note: -M represents primers applied to mice.

**Supplementary Table 3：Antibodies for immunofluorescence and**

**immunohistochemical staining**

| **Protein** | **Catalog Number** | **Company** | **Country** | **Application** |
| --- | --- | --- | --- | --- |
|  |  |  |  |  |
| PLXNC1 | HPA066899 | Sigma-Aldrich | Darmstadt, Germany | IHC (1:200) |
| Vimentin | BM0135 | Boster | Beijing, China | IF (1:100) |
| E-cadherin | sc-8426 | Santa Cruz Biotechnology | CA, USA | IF (1:50) |
| PLXNC1-M | #544232 | R&D System | MN, USA | IHC (1:50) |
| β-catenin-M | bs-1165R | Bioss Antibodies | Beijing, China | IHC (1:50) |
| PCNA-M | #2586 | Cell Signaling Technology | MA, USA | IHC (1:50) |
| CD31-M | #77699 | Cell Signaling Technology | MA, USA | IHC (1:50) |
| Vimentin-M | #5741 | Cell Signaling Technology | MA, USA | IHC (1:50) |
| TGFβ-M | sc-130348 | Santa Cruz Biotechnology | CA, USA | IHC (1:50) |
| E-cadherin-M | #3195 | Cell Signaling Technology | MA, USA | IHC (1:50) |

Note: -M represents primers applied to mice.

**Supplementary Table 4：shRNA target sequence**

| shRNA | shRNA target sequence |
| --- | --- |
| PLXNC1-shRNA | GCAGATGTCTGCCGGAATATT |
| Scramble-shRNA | CCTAAGGTTAAGTCGCCCTCG |

**Supplementary Table 5：Univariate analysis of overall survival in 566 COAD specimens**

| **Variables** | **Multivariate analysis** | |
| --- | --- | --- |
|  | **HR (95% CI)** | ***P*-value** |
| Age (≤60/60-70/＞70) | 1.576 (1.249,1.989) | <0.001* |
| Sex (Female/Male) | 1.100 (0.761,1.590) | 0.612 |
| Race (Asian/Black or African American/White) | 0.974 (0.605,1.568) | 0.913 |
| Neoplasm disease stage (Stage I/II/III/IV) | 2.152 (1.733,2.673) | <0.001* |
| Cancer metastasis stage (M0/M1/MX) | 1.565 (1.250,1.959) | <0.001* |
| Neoplasm disease lymph node stage (N0/N1/N2) | 2.004 (1.614,2.487) | <0.001* |
| Cancer tumor stage (T1/T2/T3/T4) | 2.673 (1.857,3.846) | <0.001* |
| Radiation therapy (Yes/No) | 0.228 (0.032,1.638) | 0.142 |
| Mutation count (≤90/90-150/>150) | 1.145 (0.893,1.467) | 0.285 |
| Tumor disease anatomic site (Colon/Rectum) | 0.714 (0.444,1.148) | 0.164 |
| PLXNA1 | 1.480 (1.026, 2.135) | 0.036* |
| PLXNA3 | 1.922 (1.292,2.861) | 0.001* |
| PLXNA4 | 1.626 (1.109,2.385) | 0.013* |
| PLXNB3 | 1.898 (1.309,2.751) | 0.001* |
| PLXNC1 | 2.106 (1.199,3.699) | 0.010* |
| PLXND1 | 1.906 (1.174,3.094) | 0.009* |

Univariate Cox regression model was used to evaluate the relationships of clinical variables with overall survival. COAD: Colorectal Adenocarcinoma, HR: hazard ratio, CI: confidence interval.

**Supplementary Figure 1:**


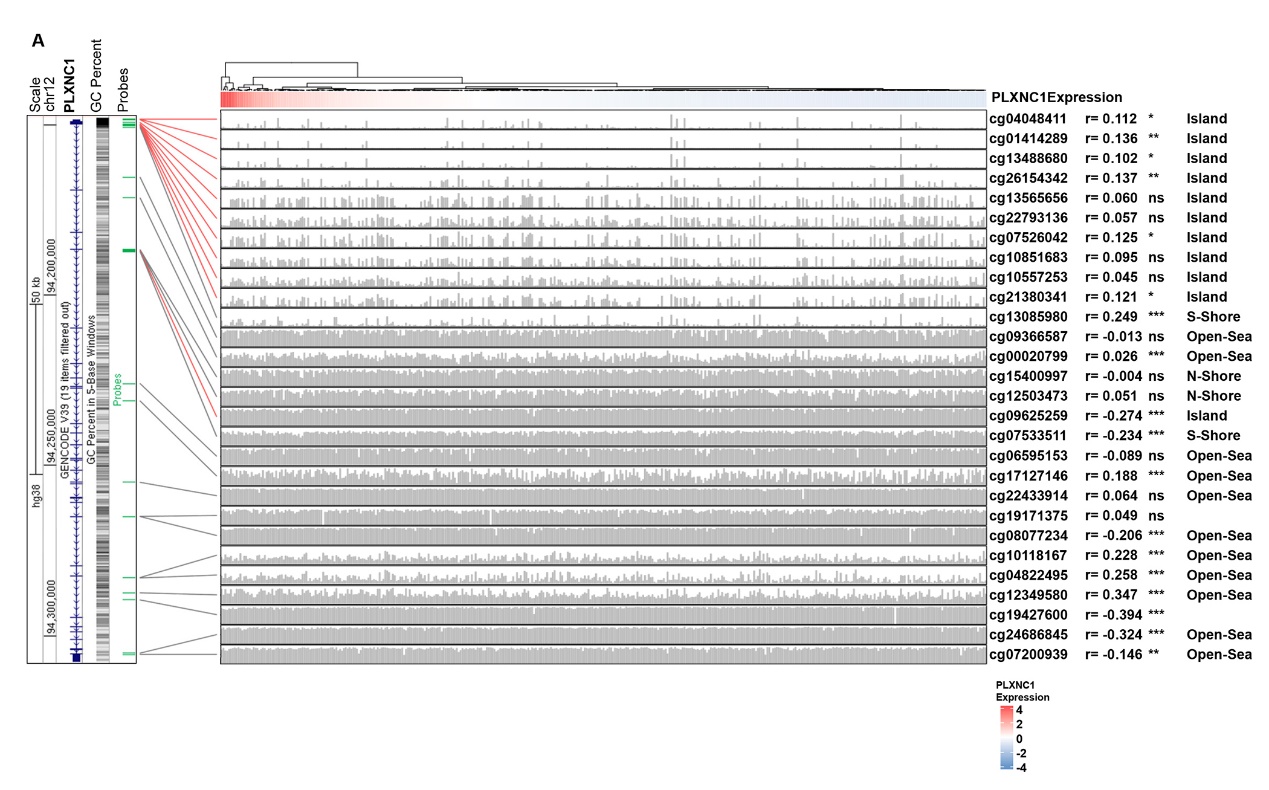


**Supplementary Figure 1.** The correlations between PLXNC1 expression and methylation probes. The genome browser on the left shows the position of methylation probes on the PLXNC1 gene. Red lines indicate probes located on CpG islands. ***, p < 0.001; **, p < 0.01; *, p < 0.05. ns, not significant.

**Supplementary Figure 2:**

**
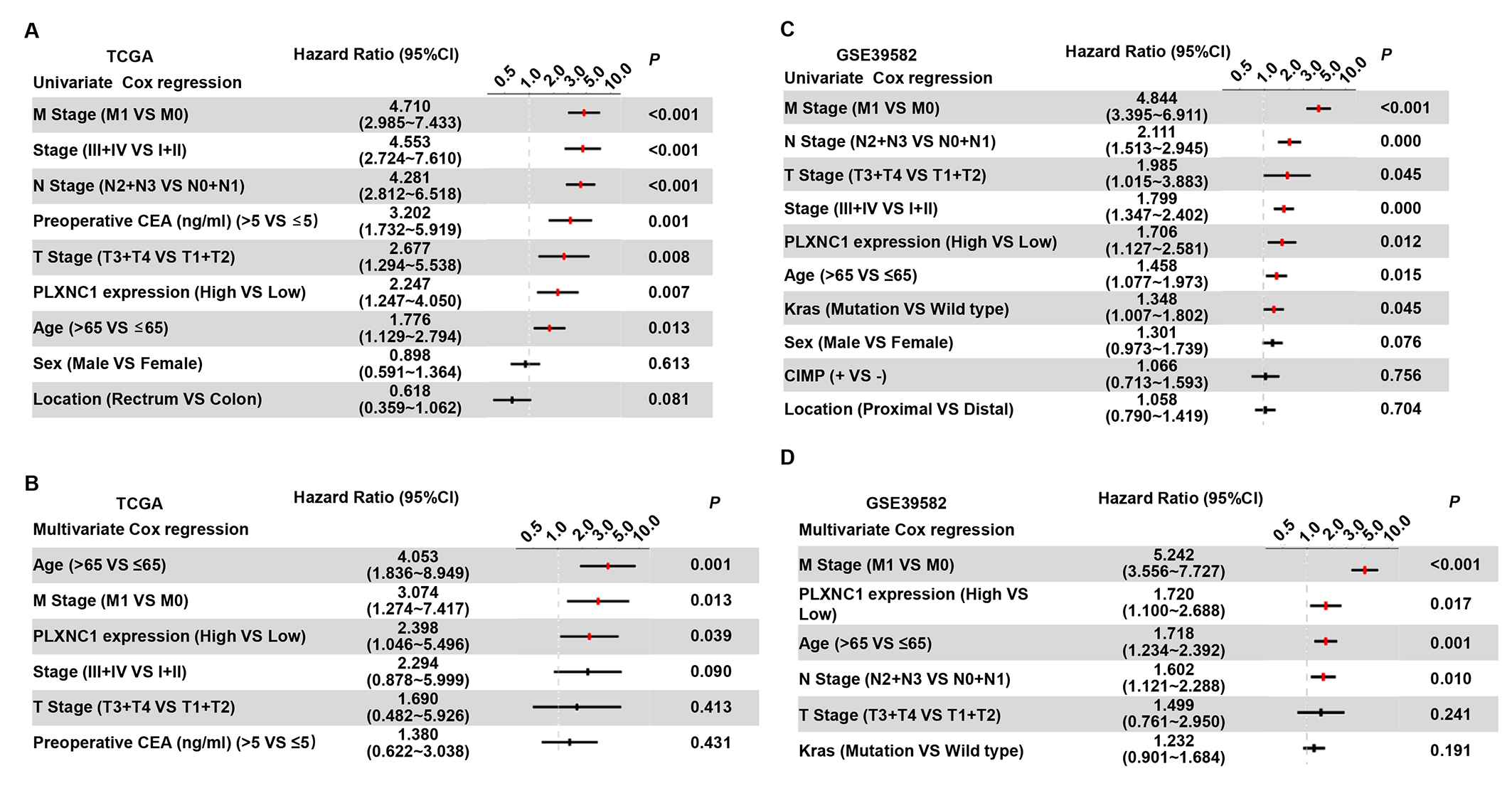
**

**Supplementary Figure 2. Prognostic value of PLXNC1 in colorectal cancer assessed by COX analysis.** Univariate Cox regression analysis of PLXNC1 expression and clinical characteristics in TCGA cohort (A) and GSE39582 cohort (C). Multivariable Cox models in (B) TCGA-COREAD and (D) GSE39582 datasets. CI: confidence interval.

**Supplementary Figure 3:**

**
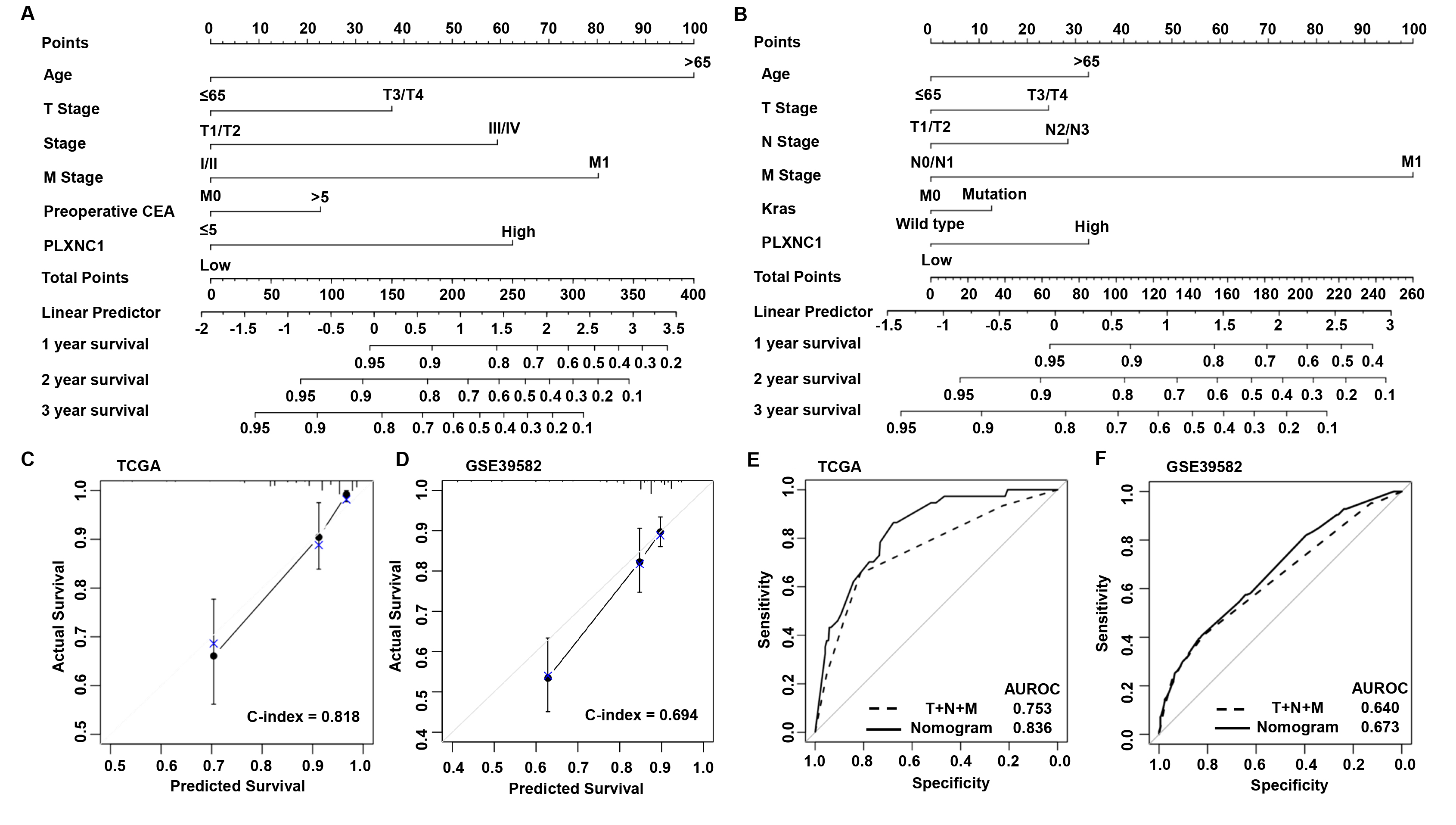
**

**Supplementary Figure 3. Nomogram for predicting overall survival in colorectal cancer patients.** Nomogram for predicting 1-, 2-, and 3-year OS of patients in (A) TCGA-COREAD and (B) GSE39582 datasets. Calibration curves show the consistency between predicted and actual 3-year OS in (C) TCGA-COREAD and (D) GSE39582 datasets. ROC curves show the predictive accuracy according to the clinical stage and the nomogram in (E) TCGA-COREAD and (F) GSE39582 datasets. AUROC: area under receiver operating characteristic.

**Supplementary Figure 4:**


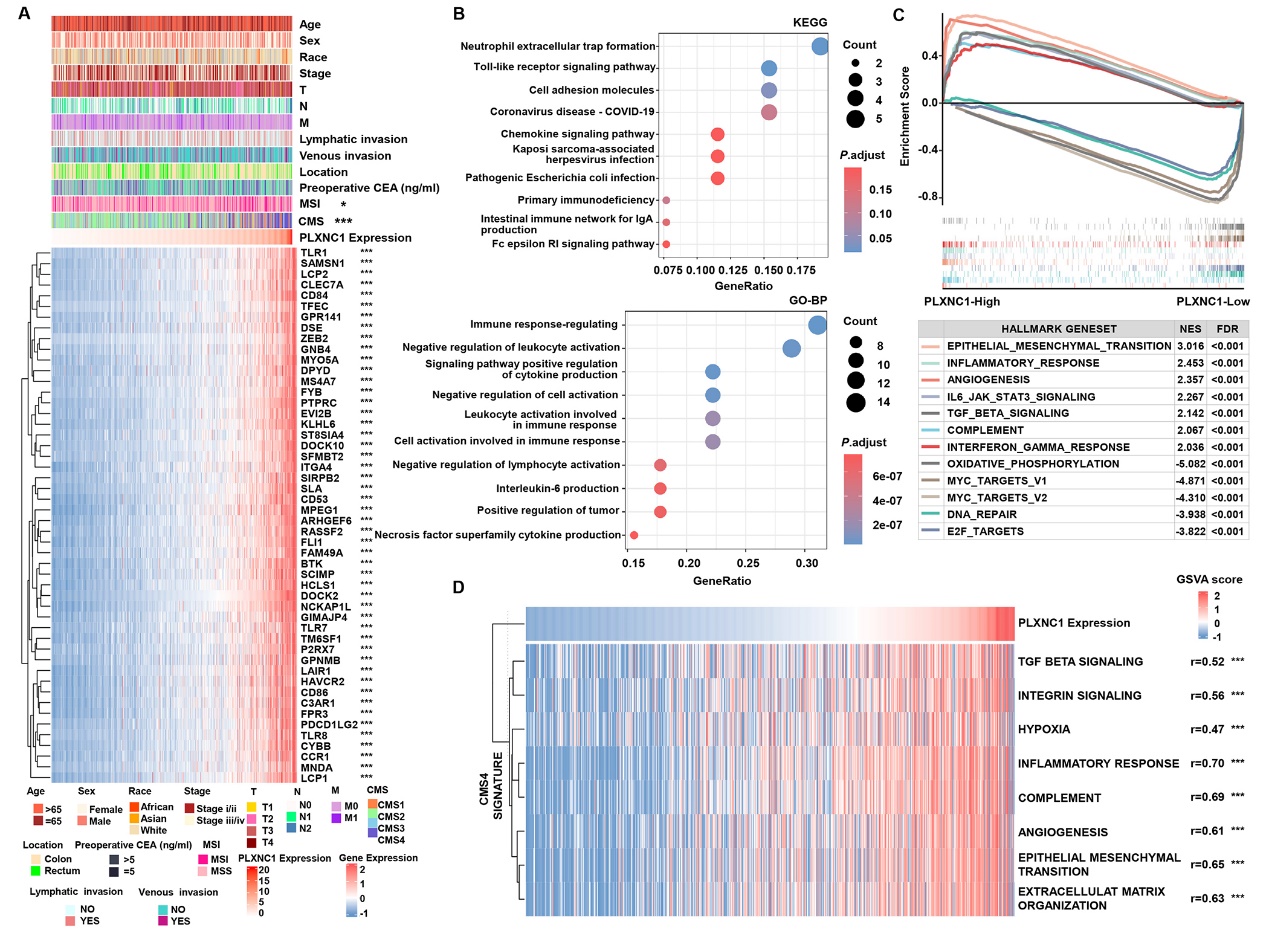
 **Supplementary Figure 4. PLXNC1 correlates with CMS4 CRC characteristics.** (A) Correlation heat map between the expression of PLXNC1 and clinical characteristics, the 50 most co-expressed genes with PLXNC1 respectively in the TCGA-COREAD cohort. (B) Enriched GO and KEGG pathways of 50 co-expressed genes. (C) GSEA enrichment plots of PLXNC1-High and PLXNC1-Low CRC patients in the TCGA cohort. (D) Correlations between PLXNC1 and the CMS4 signature in term of CRC biological processes. ***, *P* < 0.001; *, *P* < 0.05.

**Supplementary Figure 5:**

**
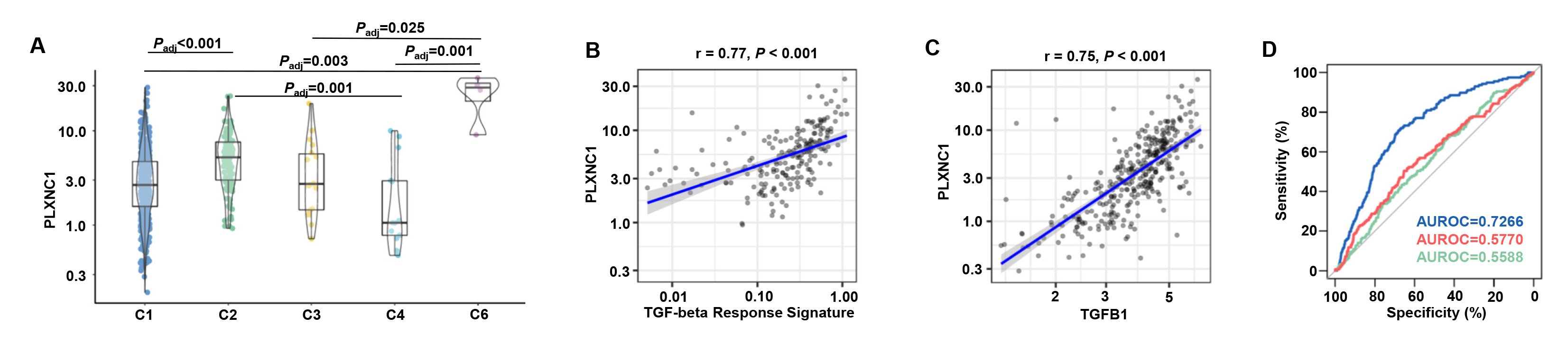
**

**Supplementary Figure 5. The correlation between PLXNC1 and immune subtypes.** (A) PLXNC1 expression in CRC patients of six immune subtypes. (B) The correlations between PLXNC1 expression and TGFB response signature or (C) TGFB1 expression in CRC patients. (D) PLXNC1 expression (blue), TGFB response signature (red), and TGFB1 expression (green) discriminate C6 subtypes from C1-5 subtypes in pan-cancer study.

**Supplementary Figure 6:**


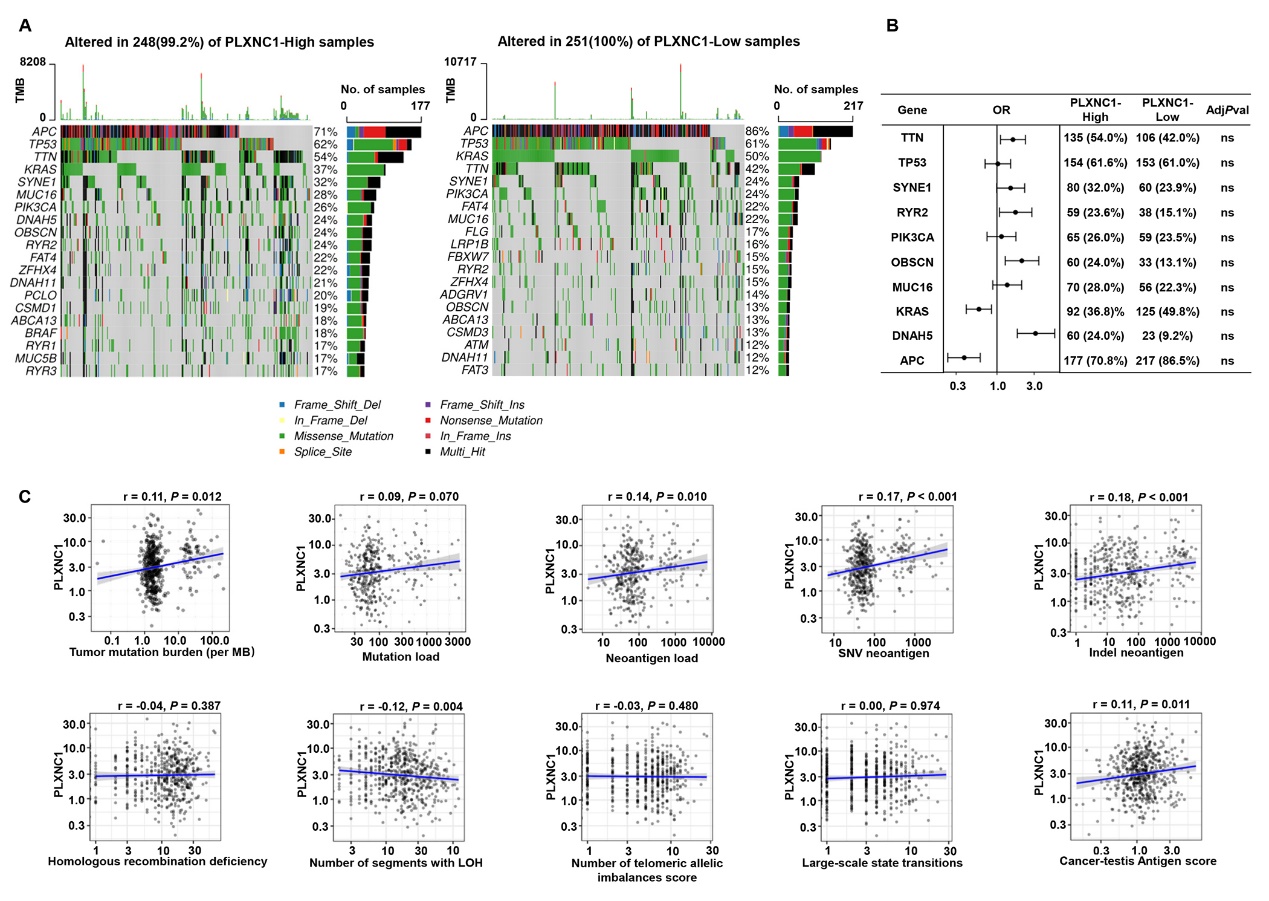


**Supplementary Figure 6.** **The correlations between PLXNC1 expression and mutation.** (A) Oncoprint plots show the mutated genes and their mutation rate in PLXNC1-High and -Low tumors. (B) The association between mutation rate and PLXNC1-High/Low groups. (C) The correlations between PLXNC1 expression and DNA damage footprint, mutation burden, and neoantigen load. ns, not significant.

**Supplementary Figure 7:**

**
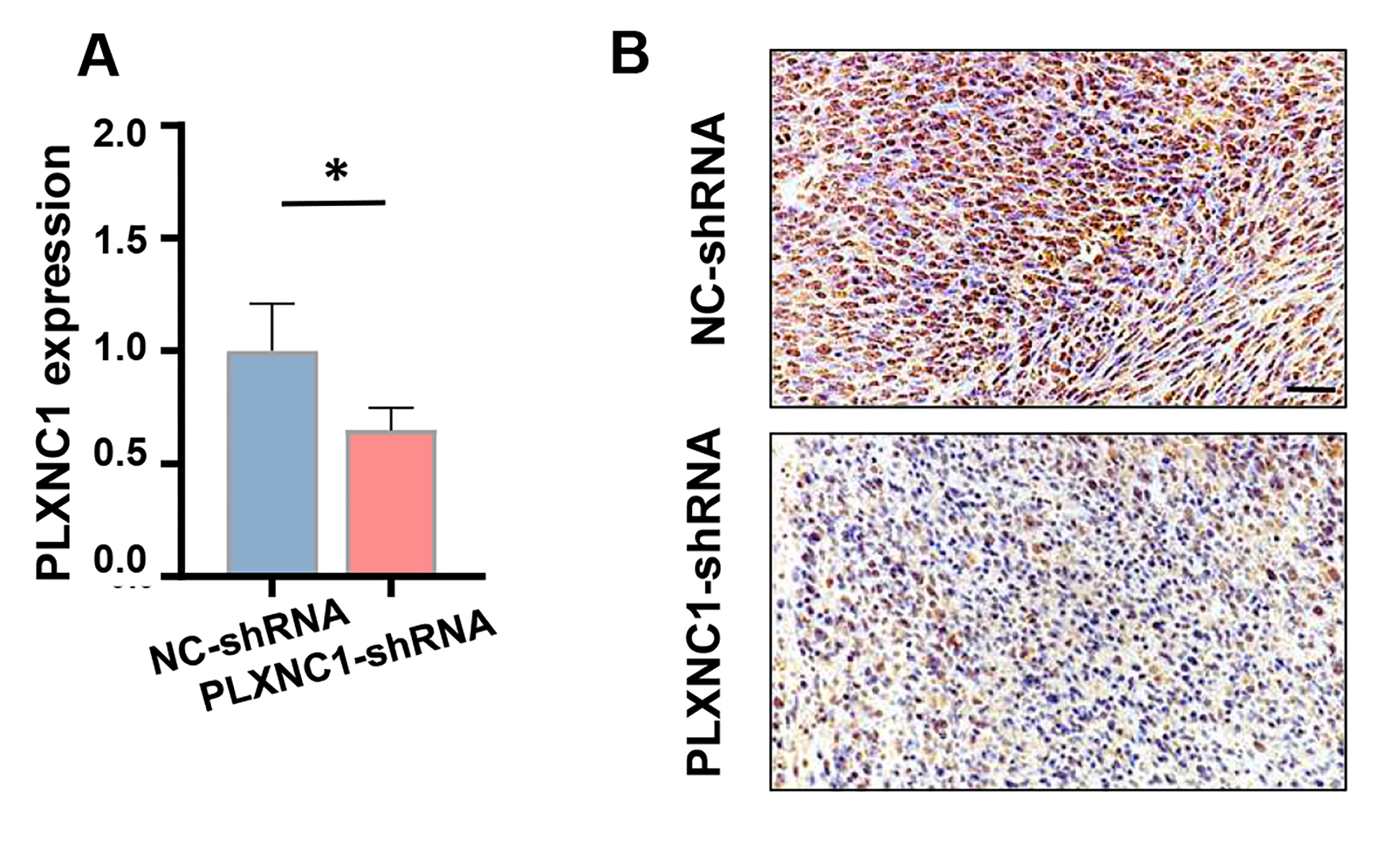
**

**Supplementary Figure 7. PLXNC1 knockdown in SL4 cells.** (A) Transcript expression levels of PLXNC1 were determined in SL4 cells infected with the lentivirus vector of sh-NC or sh-PLXNC1 by q-RT PCR. (B) Immunohistochemistry of tumor tissues from WT mice after subcutaneous inoculation of SL4 cells infected with the control vector or sh-PLXNC1 vector was used to detect the protein expression of PLXNC1. Note: Scale bars=75 μm. **P* < 0.05

**Supplementary Figure 8:**

**
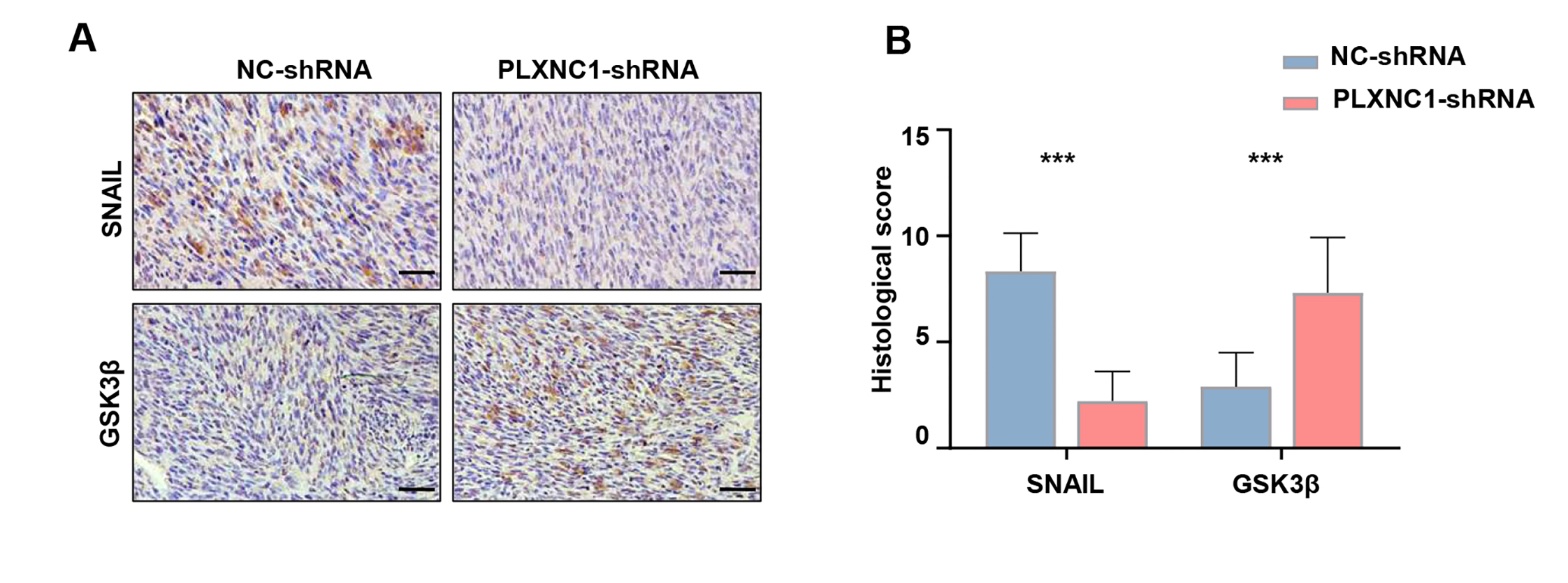
**

**Supplementary Figure 8. Immunohistochemical staining showing expression of EMT markers in subcutaneous tumor tissues.** (A) Representative immunohistochemical staining picture and quantification analysis shows the protein expression levels of SNAIL and GSK3β. (B) Quantification of immunohistochemical staining in subcutaneous tumor tissues from sh-NC and sh-PLXNC1 groups. ****P* < 0.001.

**Supplementary Figure 9:**

**
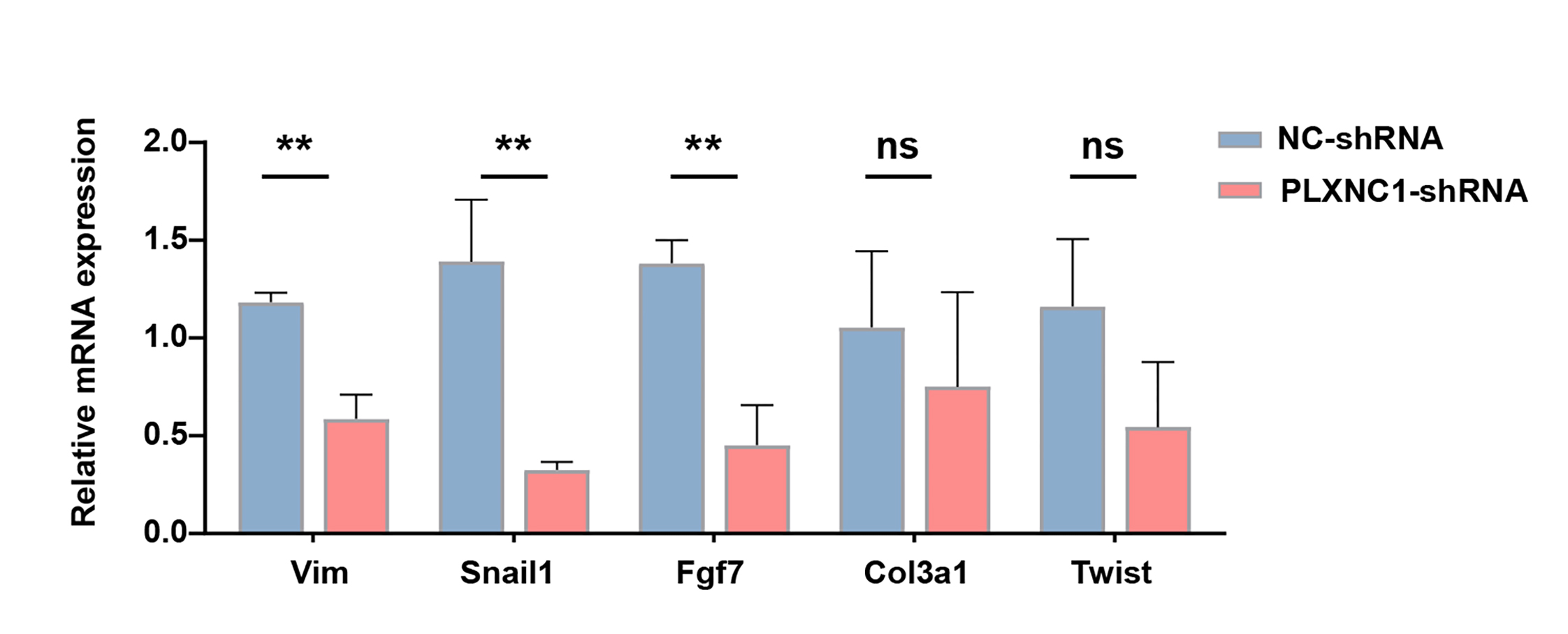
**

**Supplementary Figure 9.** The relative mRNA expression of EMT-related genes in liver metastasis tumor tissues from sh-NC and sh-PLXNC1 groups, detected by q-RT PCR analysis. ***P* < 0.01; ns, not significant.

**Supplementary Figure 10:**

**
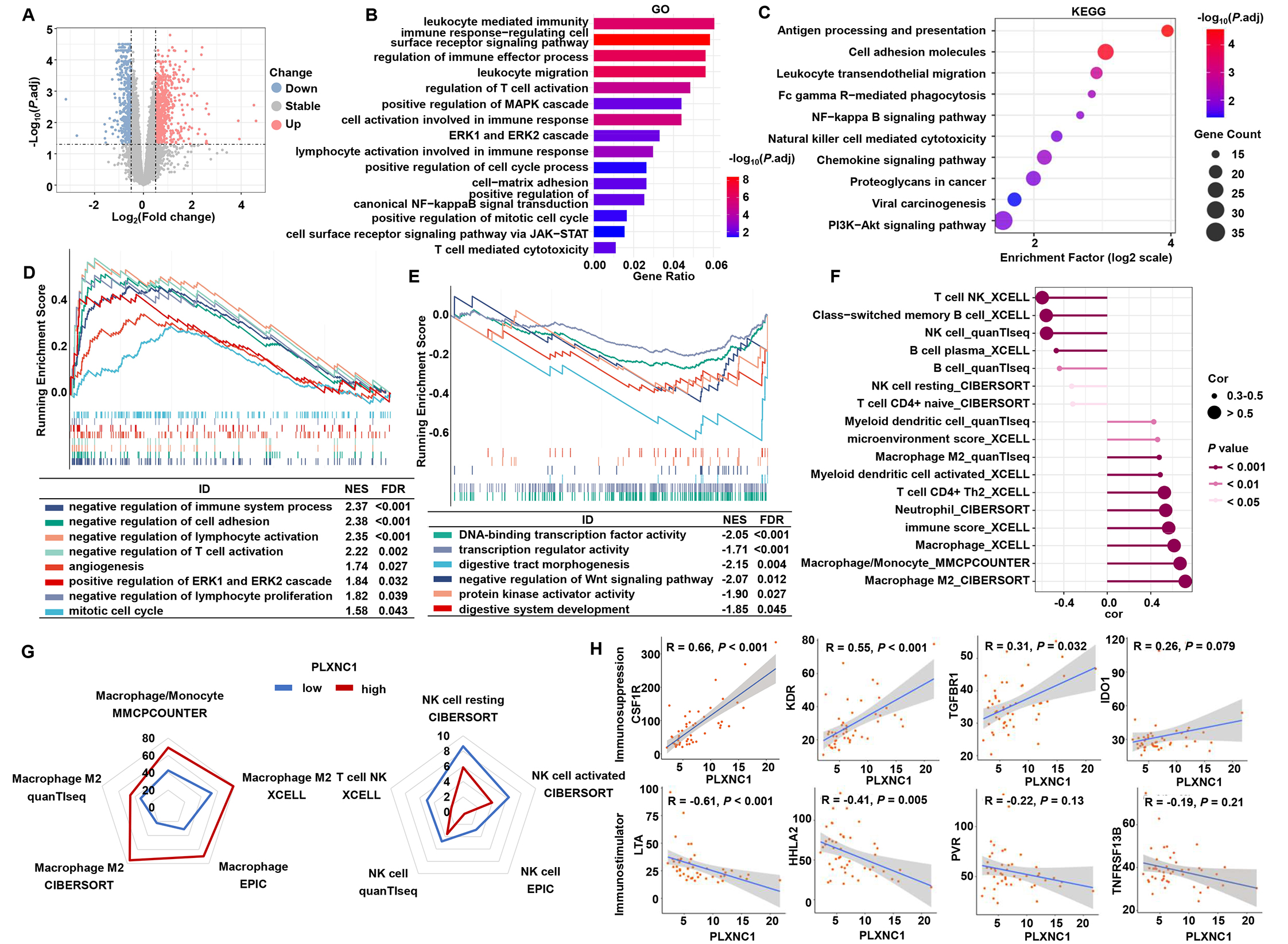
**

**Supplementary Figure 10. PLXNC1 Expression Associates with an Immunosuppressive Microenvironment in Colorectal Cancer Liver Metastasis.** (A) Volcano plot of differentially expressed genes between PLXNC1-high and PLXNC1-low groups (stratified by median PLXNC1 expression) in GSE41258, with thresholds of adjusted *P* value < 0.05 and |log₂FC| > 0.5. (B) GO enrichment of differentially expressed genes between PLXNC1-high and PLXNC1-low groups. (C) KEGG enrichment of differentially expressed genes between PLXNC1-high and PLXNC1-low groups. GSEA enrichment plots of liver metastasis tumor in the PLXNC1-high (D) and PLXNC1-low (E) groups. (F) Correlation between PLXNC1 expression and immune cell infiltration evaluated through multiple algorithms. (G) Radar map showing Macrophages and NK cells infiltration in the PLXNC1-high and PLXNC1-low groups. (H) Correlation of PLXNC1 expression with immunosuppressive and immunosimulative markers in metastasis tumor tissues from sh-NC and sh-PLXNC1 groups.

**Supplementary Figure 11:**

**
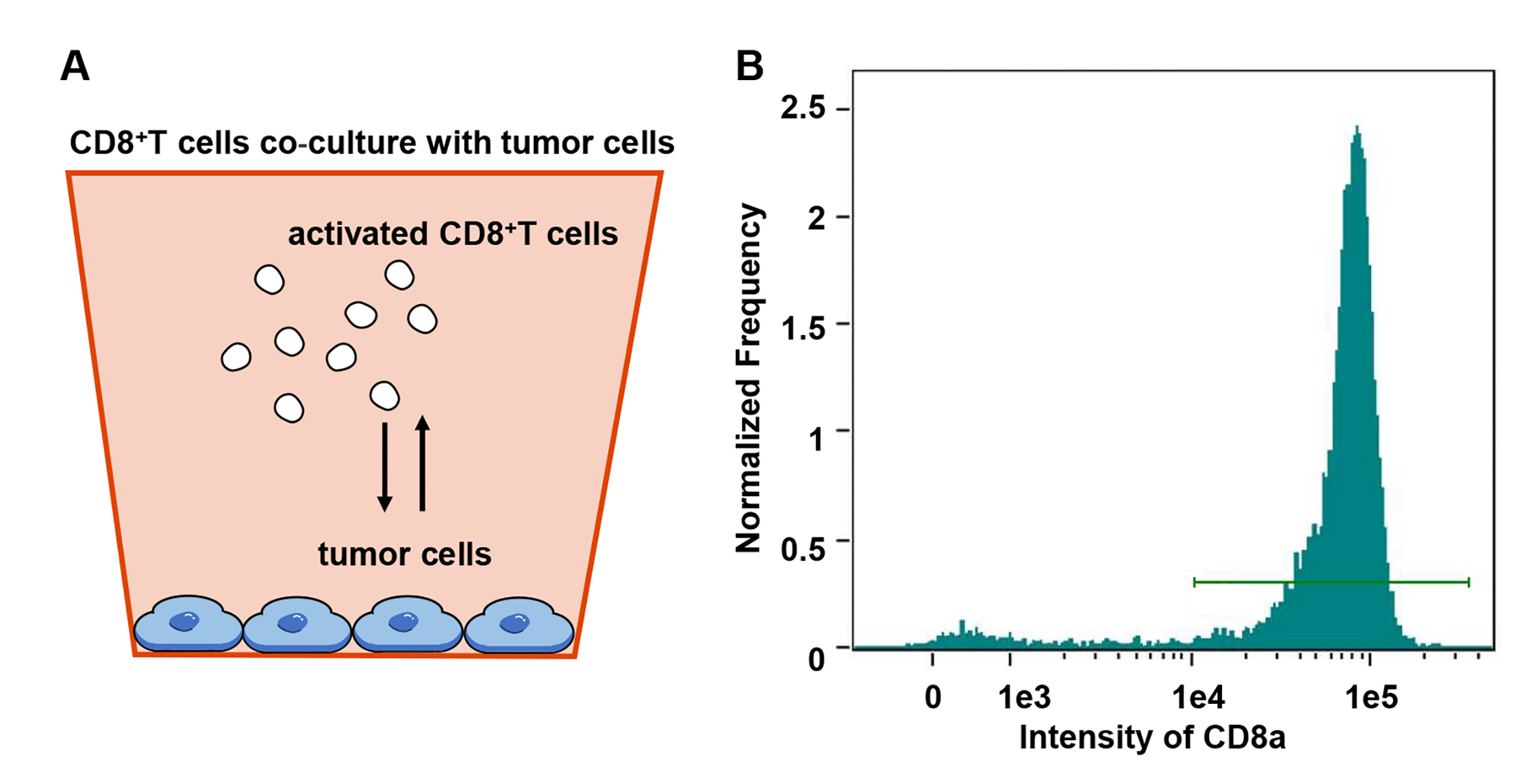
**

**Supplementary Figure 11. Establishment and validation of CD8⁺ T Cell-SL4 coculture system** (A) The schematic diagram illustrates the co-culture system of CD8+T cell and SL4 cell. (B) Representative flow cytometry plot showing CD8+ T cell population after isolation.

**Supplementary Figure 12:**


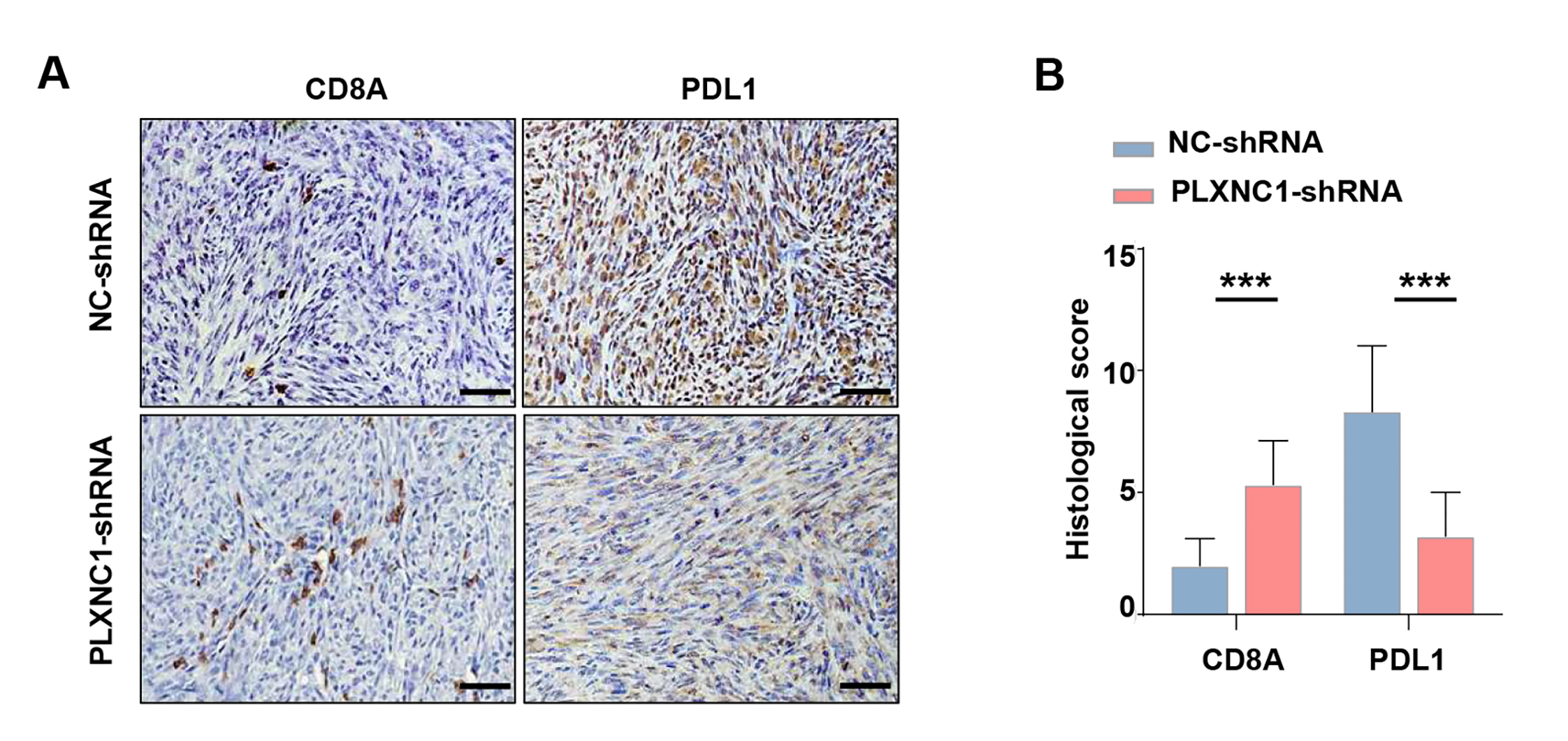


**Supplementary Figure 12. Functional effects of PLXNC1 silencing on tumor immune microenvironment.** (A) Immunohistochemical staining showing expression of immune markers in liver metastasis tumor tissues from sh-NC and sh-PLXNC1 groups. (B) Quantification of immunohistochemical staining in liver metastasis tumor tissues from sh-NC and sh-PLXNC1 groups. ***, *P* < 0.001.

**Supplementary Figure 13:**

**
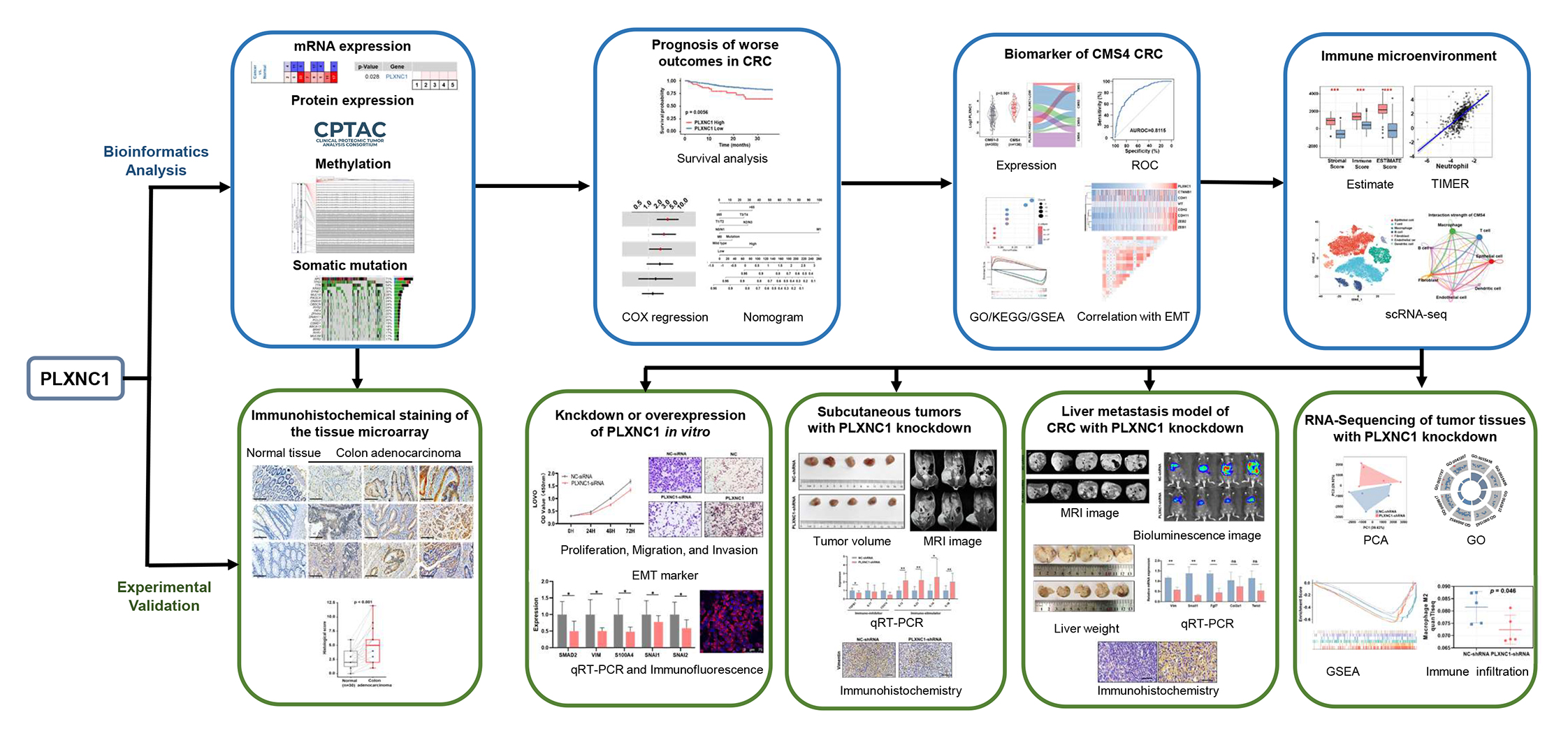
**

**Supplementary Figure 13. The workflow of the present study.**
